# EPICERTIN, an engineered variant of cholera toxin B subunit, promotes survival and a pro-remodeling macrophage phenotype for mucosal healing in colitis

**DOI:** 10.1016/j.mucimm.2026.01.013

**Published:** 2026-02-04

**Authors:** Noel Verjan Garcia, Jimmy Fernando Cifuentes, Micaela A. Reeves, Jae Yeon Hwang, Juw Won Park, Susan Galandiuk, Nobuyuki Matoba

**Affiliations:** a Center for Predictive Medicine, University of Louisville, Louisville, KY, USA; b Department of Pharmacology and Toxicology, University of Louisville School of Medicine, Louisville, KY, USA; c UofL Health – Brown Cancer Center, University of Louisville, Louisville, KY, USA; d Department of Surgery, Price Institute of Surgical Research, University of Louisville, Louisville, KY, USA; e The Center for Integrative Environmental Health Sciences Biostatistics and Informatics Facility Core, University of Louisville, Louisville, KY, USA; f Brown Cancer Center Bioinformatics Core, Department of Medicine, University of Louisville, KY, USA; g KY INBRE Bioinformatics Core, University of Louisville, Louisville, KY, USA

**Keywords:** M2-like macrophage, mucosal healing, cholera toxin B subunit, colitis, apoptosis

## Abstract

EPICERTIN, a modified cholera toxin B subunit (CTB), facilitates mucosal healing in preclinical colitis models, but its anti-inflammatory mechanisms remain unclear. Here, we investigated EPICERTIN’s effects on macrophages. In a dextran sulfate sodium-induced colitis mouse model, oral administration of EPICERTIN reduced neutrophil infiltration and increased CX3CR1^+^MHCII^lo/−^ (M2-like) over CX3CR1^+^MHCII^hi^ (M1-like) macrophages in the colon lamina propria. This was concurrent with upregulation of colony-stimulating factor 2 (*Csf2*) and growth factors (*Egf*, *TgfA*, *Fgf*, *Pdgf*) involved in mucosal remodeling. Similarly, in colon tissue from a human with active colitis, EPICERTIN significantly upregulated *CSF2* and tissue repair-associated genes while downregulating proinflammatory genes (*IL1B*, *IL6ST*). In vitro, EPICERTIN promoted macrophage survival under serum-free conditions, whereas CTB induced apoptosis in murine RAW264.7 cells, peritoneal macrophages, and human THP-1 cells. Remarkably, EPICERTIN protected macrophages from apoptosis induced by chemical ER-stressors or lipopolysaccharides. Additionally, EPICERTIN downregulated cell surface molecules HLA-DR, CD14, CD80, and CD86 in THP-1 cells and modestly upregulated chemokines and proinflammatory cytokines genes as well as *TGFB1* in human PBMC-derived macrophages. In contrast, CTB strongly increased proinflammatory genes and activation markers. These findings indicate that EPICERTIN promotes macrophage homeostasis by inducing a less inflammatory, pro-remodeling phenotype, whereas CTB may trigger activation-induced cell death.

## INTRODUCTION

Inflammatory bowel disease (IBD) primarily comprises ulcerative colitis (UC) and Crohn’s disease (CD), with the former characterized by mucosal inflammation of the colon and rectum, whereas the latter is manifested by transmural inflammation of any region of the gastrointestinal tract.^1,2^ IBD is multifactorial in nature, and current therapies are effective only in a subset of patients.^3,4^ Others remain at increased risk of developing colorectal cancer, highlighting the urgent need for novel prevention and treatment options promoting mucosal healing of IBD patients.^5^.

Mucosal healing is a complex pathophysiological process involving distinct types of cells, including epithelial, stromal and immune cells that converge in the secretion of cytokines and growth factors to regenerate damaged tissue. This may lead to complete restitution of the colonic mucosa with the absence of erosions, ulcers and signs of inflammation.^6^ Intestinal macrophages expressing the fractalkine (FKN) receptor CX3C-chemokine receptor 1 (CX3CR1) are strategically located near the gut epithelial barrier to maintain tissue homeostasis,^7,8^ and mice deficient in CX3CR1 develop more severe DSS-colitis with increased secretion of pro-inflammatory cytokines (TNFα, IL-1β, IL-6).^9^ Thus, exploring novel mechanisms to modulate macrophage function and reduce their inflammatory phenotype might be important in IBD treatment.

EPICERTIN is a recombinant variant of the non-toxic and strong immunogen cholera toxin B subunit (CTB), engineered with an Asn4 → Ser substitution and a C-terminal SEKDEL hexapeptide for endoplasmic reticulum (ER) retention.^10,11^ Recombinant CTB is used as an active component of the Dukoral oral cholera vaccine and has been utilized in vaccine development^12–15^ and in the treatment of colitis in mice^16^ and CD in humans.^17^ EPICERTIN, originally created as an alternative subunit cholera vaccine,^18^ retains mucosal immunogenicity and stimulates antibody production without affecting its mucosal healing effects.^19^ Both CTB and EPICERTIN bind to GM1 ganglioside at the cell surface and are transported retrogradely to the ER;^20^ however, only EPICERTIN contains an ER retention motif to interact with KDEL receptors (KDELR), prolonging its retention time to induce an IRE1-XBP1-mediated adaptive unfolded protein response.^21^ This adaptive UPR leads to transforming growth factor beta (TGF-β) signaling and results in mucosal healing, epithelial restitution, and reduced inflammation in acute and chronic colitis mouse models.^19,21,22^ TGF-β signaling is known to mediate wound healing of the intestinal mucosa^23^ and of the skin^24^. While EPICERTIN’s mucosal healing effects involve epithelial restitution and barrier recovery to stop the ingress of insulting agents to the colon lamina propria (CLP), its immunomodulatory potential has yet to be demonstrated. In particular, modulation of macrophage functions may be part of EPICERTIN’s mucosal healing effects in colitis, as these cells are essential in antigen presentation, tissue remodeling, and clearing infection. EPICERTIN administered orally localizes in colon crypt epithelial cells,^25^ but its interaction with immune cells has not been explored.

**Fig. 1.**
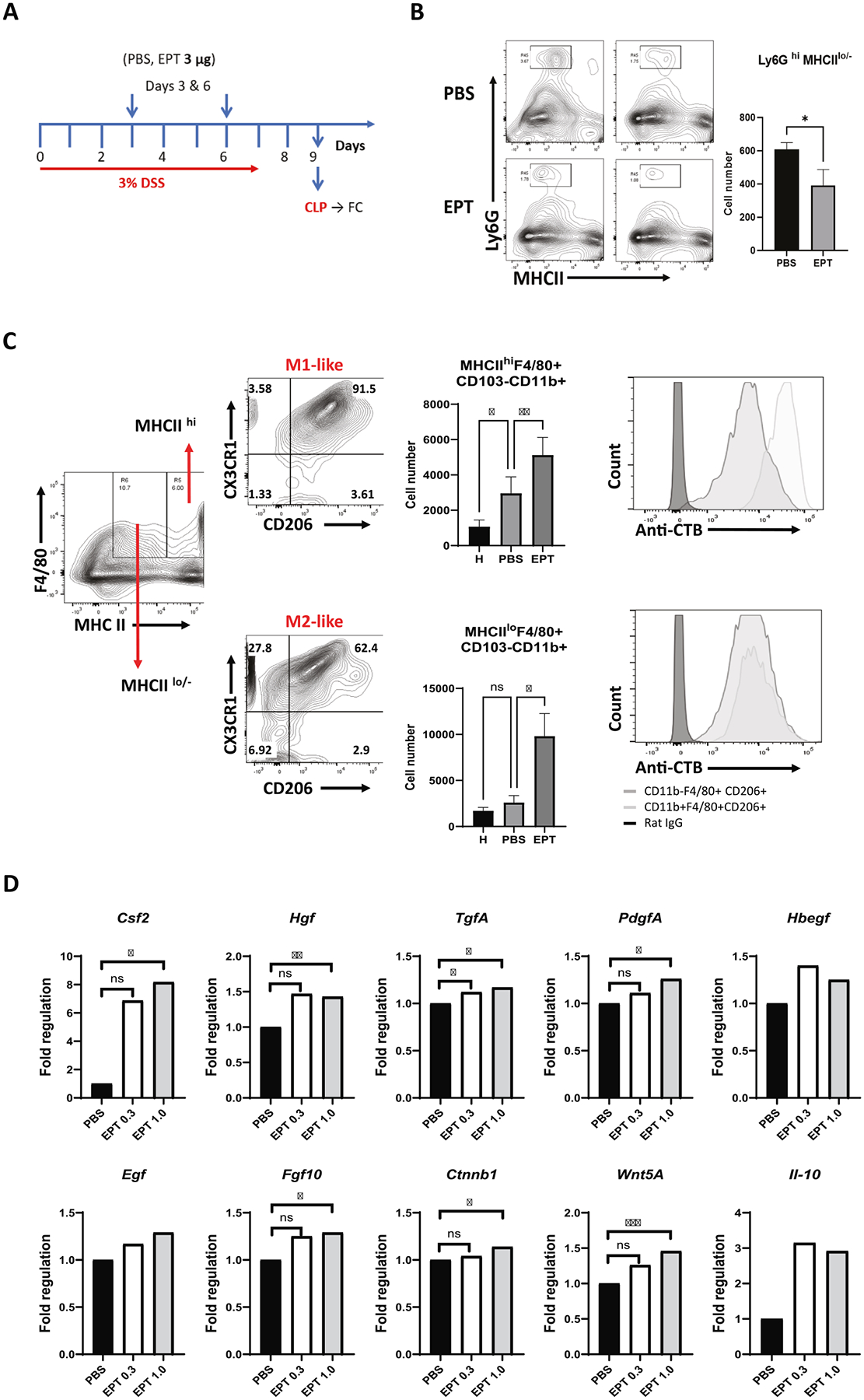
EPICERTIN suppresses neutrophil infiltration and promotes re-epithelialization and macrophage survival in the colon lamina propria of DSS colitis mice. A. Experimental design of colitis induced by 3% DSS in drinking water for seven days. Mice were untreated (H, healthy) or treated with PBS or EPICERTIN orally at days 3 and 6 of the DSS cycle, and CLP and MLNs leukocytes were isolated on day 9 for flow cytometry. B. EPICERTIN reduced neutrophil infiltration into the CLP and (C) significantly (p < 0.01 and p < 0.05) recruited both M1-like (CD11b^+^F4/80^+^MHCII^hi^CD103^−^) and M2-like (CD11b^+^F4/80^+^MHCII^lo/−^CD103^−^) macrophages that also expressed CX3CR1 and CD206 markers (C). Absolute numbers of M2-like macrophages were nearly twice those of M1-like macrophages. Histograms show higher EPICERTIN binding to M1-like macrophages. D. Oral treatment of DSS colitis mice with EPICERTIN (0.3 or 1.0 μg) on days 3 and 6 revealed a dose-dependent significant increase in transcription of colony stimulating factor 2 (*Csf2*) and other cell growth factors involved in wound healing. Gene expression is presented as Fold Regulation, calculated using the ΔΔCT method according to the Qiagen RT^2^ Profiler PCR array analysis workflow. For each gene, one ΔCT value was generated per biological replicate (n = 3 per treatment group), normalized to the panel’s designated housekeeping genes. Statistical significance was determined using an unpaired two-tailed *t*-test on the individual 2^−ΔCT^ values from the three biological replicates. Because Fold Regulation is a ratio derived from group-averaged 2^−ΔCT^ values (ΔΔCT method), standard deviation bars cannot be meaningfully applied and therefore not shown, consistent with manufacturer recommendations. A *t*-test (B, D) or One-way ANOVA with Bonferroni’s multiple comparison test was used to compare between groups (C). Significant differences are indicated with asterisks (*p < 0.05, **p < 0.01, ***p < 0.001, ****p < 0.0001).

**Fig. 2.**
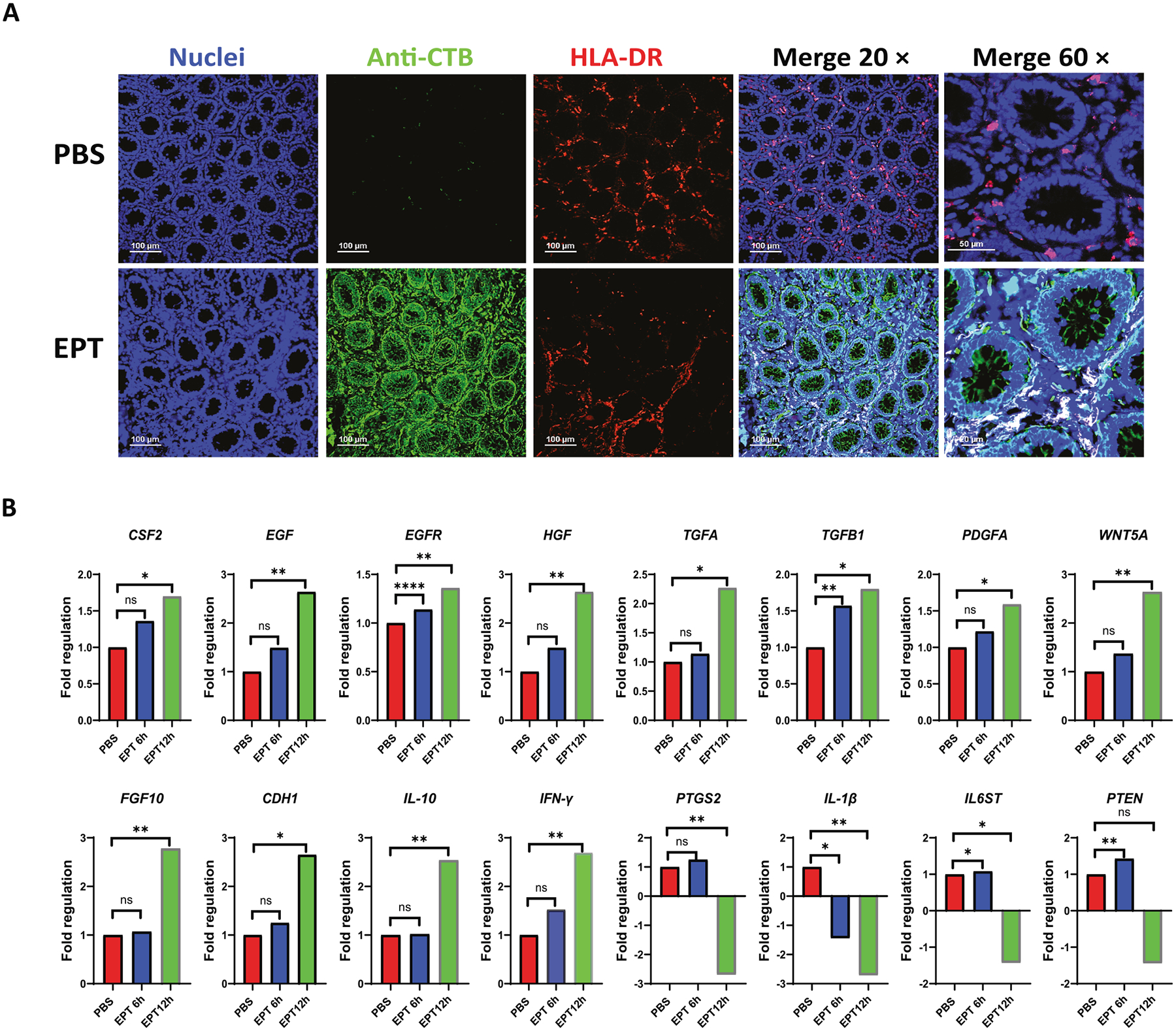
EPICERTIN binds to human colitis tissue and modulates wound-healing gene expression in human colon explants. A. Immunohistochemical detection of EPICERTIN binding in human colon tissue from patients with active colitis. A total of seven independent colon explant specimens were processed, and one representative image is shown. Tissue sections were incubated with EPICERTIN followed by anti-CTB antibody staining; HLA-DR^+^ leukocytes are indicated as reference for immune cell localization. B. Gene expression analysis of wound-healing-associated factors in human colon tissue using the RT^2^ Profiler Human Wound Healing PCR Array. Colon explants were obtained from three independent donors (two with active colitis and one with colorectal cancer). Due to substantial variability in cancer-derived tissues, likely reflecting prior therapeutic exposure and tumor-associated remodeling, the results from one representative active colitis donor with intact tissue architecture are shown. Expression values are presented as Fold Regulation, calculated using the ΔΔCT method according to the Qiagen analysis workflow. For each biological sample, one ΔCT value was generated per gene, normalized to the panel’s housekeeping genes. Statistical significance for each gene was determined using an unpaired two-tailed *t*-test on the individual 2^−ΔCT^ values from the three biological replicates (three independent tissue samples per gene). Because Fold Regulation is derived from group-averaged 2^−ΔCT^ values, standard deviation bars cannot be meaningfully applied and are therefore not shown. EPICERTIN treatment revealed a significant time-dependent induction of *CSF2* and other epithelial and stromal cell growth factor genes while suppressing inflammatory related genes. Significant differences are indicated with asterisks (*p < 0.05, **p < 0.01, ***p < 0.001,****p < 0.0001).

**Fig. 3.**
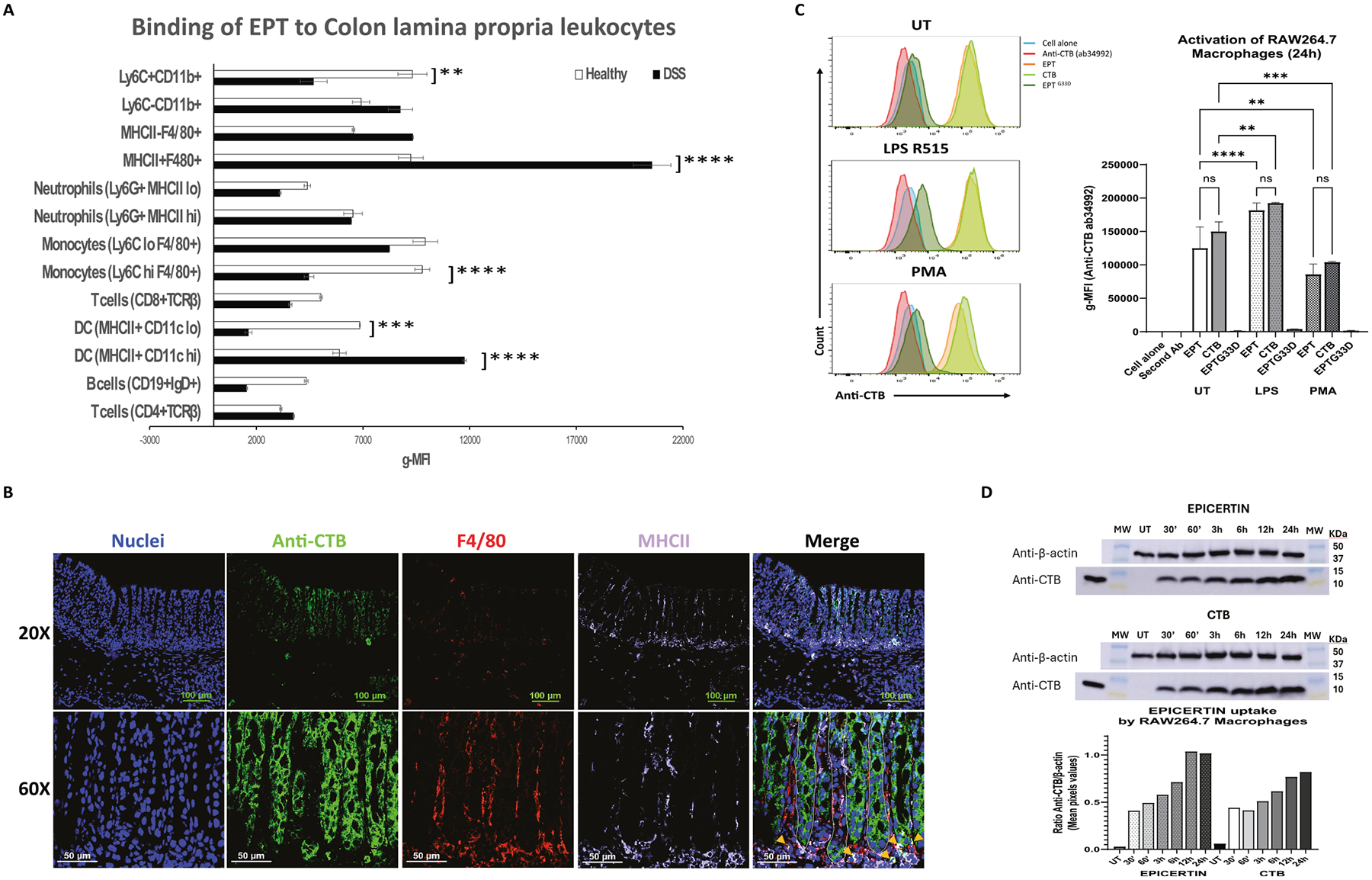
EPICERTIN and CTB binding to MHCII^+^ macrophages increases upon cell activation or inflammation. A. Flow cytometry analysis of EPICERTIN binding to lamina propria leukocytes isolated from healthy or inflamed (DSS colitis) mouse colon. Cells were gated using the same gating sequence applied in Supplemental Fig. 1A, beginning with singlets → live cells → CD45^+^ leukocytes, followed by identification of the indicated immune cell populations using established marker combinations. Cells were incubated with EPICERTIN at 4 °C for 1 h to assess surface binding only, followed by staining with anti-CTB (9F9C7-FITC) antibody. B. IHC of colon tissue from DSS-colitis mice treated with EPICERTIN as described above. EPICERTIN is localized mainly in colon crypt epithelial cells, with F4/80^+^MHCII^+^ macrophages located in the colonic lamina propria just beneath the base of colonic crypts, and around the length of the crypt, as indicated by orange arrowheads in the merged image. C. Flow cytometry analysis of CTB, EPICERTIN (labeled EPT on x-axis) and EPT^G33D^ binding to RAW264.7 macrophages under steady state conditions (UT) or at 24 h after activation by *E. coli* LPS R515 (1 μg/mL) or PMA (100 ng/mL). Background geometric mean fluorescence intensity (g-MFI) of anti-CTB (ab34992) rabbit polyclonal antibody detected with AF-488-conjugated goat-anti-rabbit Ig-G were subtracted from g-MFI of bound proteins. D. Western blot analysis of total cell-associated EPICERTIN or CTB (surface-bound + internalized) in RAW264.7 macrophages. EPICERTIN or CTB uptake was assessed after incubation at 37 °C, followed by cell lysis and detection with anti-CTB antibody. β-actin served as a loading control. One-way ANOVA with Bonferroni’s multiple comparison test was used to compare between groups (A, C). Significant differences are indicated with asterisks (*p < 0.05, **p < 0.01, ***p < 0.001, ****p < 0.0001).

**Fig. 4.**
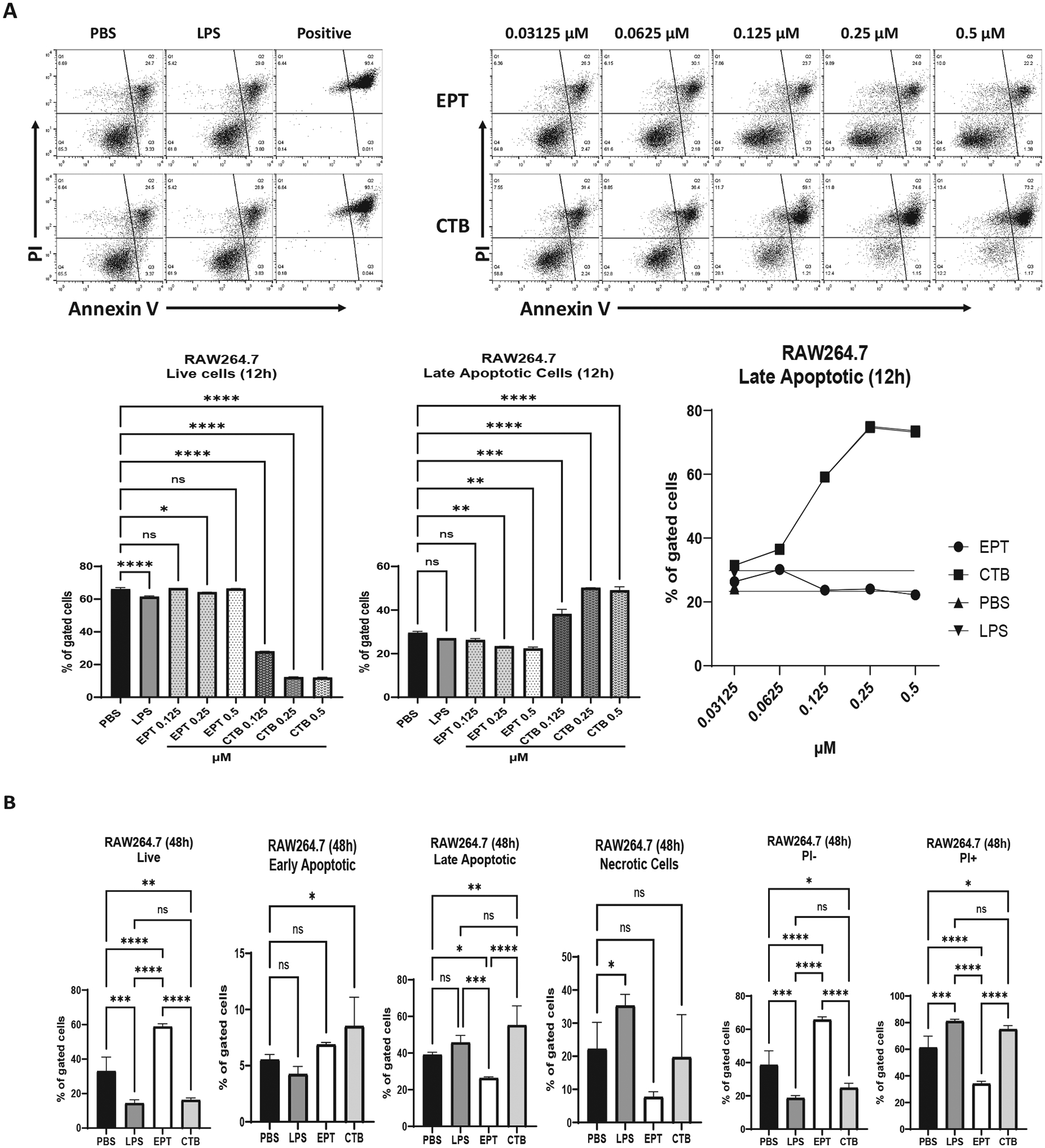
EPICERTIN promotes survival whereas CTB induces apoptosis in RAW264.7 macrophages in a dose dependent manner. RAW264.7 macrophages were treated with PBS, LPS R515 (1 μg/mL), and EPICERTIN (EPT on x-axis) or CTB (0.03125 μM – 0.5 μM) in serum-free DMEM containing 0.1% BSA for 12 h (**A**) and up to 48 h (**B**) then stained with Annexin V and PI for the presence of apoptotic cells. **A.** Control positive apoptotic cells were incubated at 56 °C for 10 min. CTB, but not EPICERTIN, induced a dose-dependent and significant (*p* < 0.0001) increase in late apoptotic cells and total PI^+^ cells at doses of 0.125, 0.25, and 0.5 μM, whereas EPICERTIN maintained cell viability at the same dose levels. The effects of EPICERTIN or CTB at doses of 0.03125 and 0.0625 μM were not significantly different to the PBS or LPS controls at 12 h. **B**. RAW264.7 macrophages were treated with PBS, LPS R515 (1 μg/mL), EPICERTIN or CTB (0.5 μM) for 48 h in serum-free conditions. Apoptotic cells increased over time in CTB and LPS treated RAW264.7 macrophages, whereas EPICERTIN significantly (*p* < 0.0001) promoted survival and reduced late apoptotic cells compared to the PBS treated control cells. One-way ANOVA with Bonferroni’s multiple comparison test was used to compare between groups. Significant differences are indicated with asterisks (*p < 0.05, **p < 0.01, ***p < 0.001, ****p < 0.0001). A representative experiment from more than five is shown in plots and each data point represents the mean ± SD of duplicate (**A**) or triplicate (**B**) samples.

**Fig. 5.**
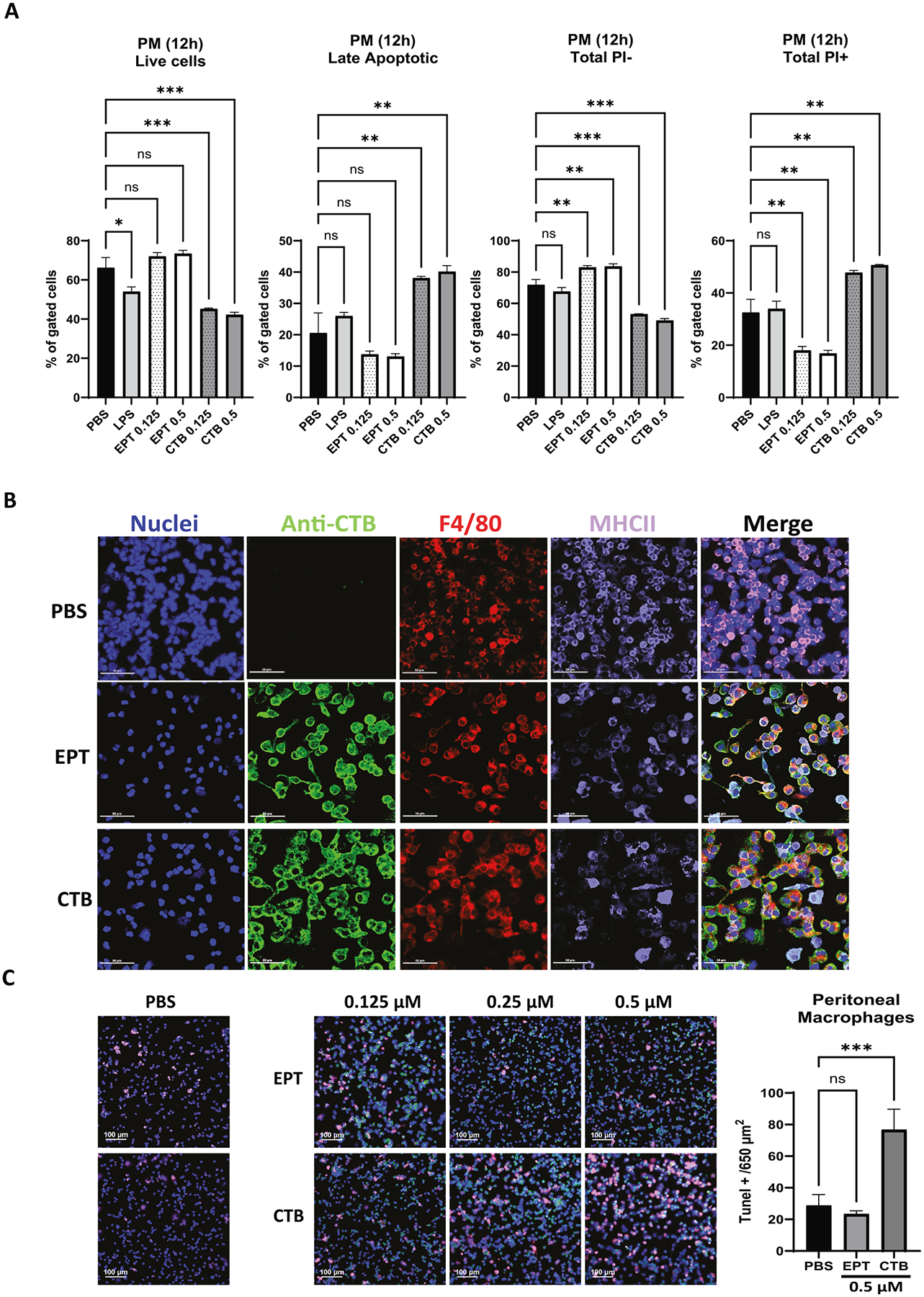
EPICERTIN promotes the survival of resident peritoneal macrophages whereas CTB induces morphological changes and DNA fragmentation features of apoptosis. **A.** Peritoneal macrophages (PM) from healthy C57BL/6 mice were treated with a low (0.125 μM) and a high (0.5 μM) dose of EPICERTIN or CTB for 12 h in serum-free medium. EPICERTIN promoted survival of primary peritoneal macrophages with no significant differences compared to PBS control, whereas CTB significantly decreased live cells and increased late apoptotic cells or total PI^+^ cells. **B**. CTB, but not EPICERTIN, induced prominent morphological changes in peritoneal macrophages. EPICERTIN-treated peritoneal macrophages appeared rounded or elongated with smooth surfaces, whereas CTB-treated macrophages appeared swollen with irregular surfaces and were severely vacuolated. **C**. CTB, but not EPICERTIN, significantly increased TUNEL-positive apoptotic macrophages visualized by confocal laser scanning microscopy. One-way ANOVA with Bonferroni’s multiple comparison test was used to compare between groups (**A, C**). Significant differences are indicated with asterisks (*p < 0.05, **p < 0.01, ***p < 0.001, ****p < 0.0001). A representative experiment from two is shown in **A** and each data point represents the mean ± SD of duplicate samples. One individual experiment is shown in **B** or **C**.

**Fig. 6.**
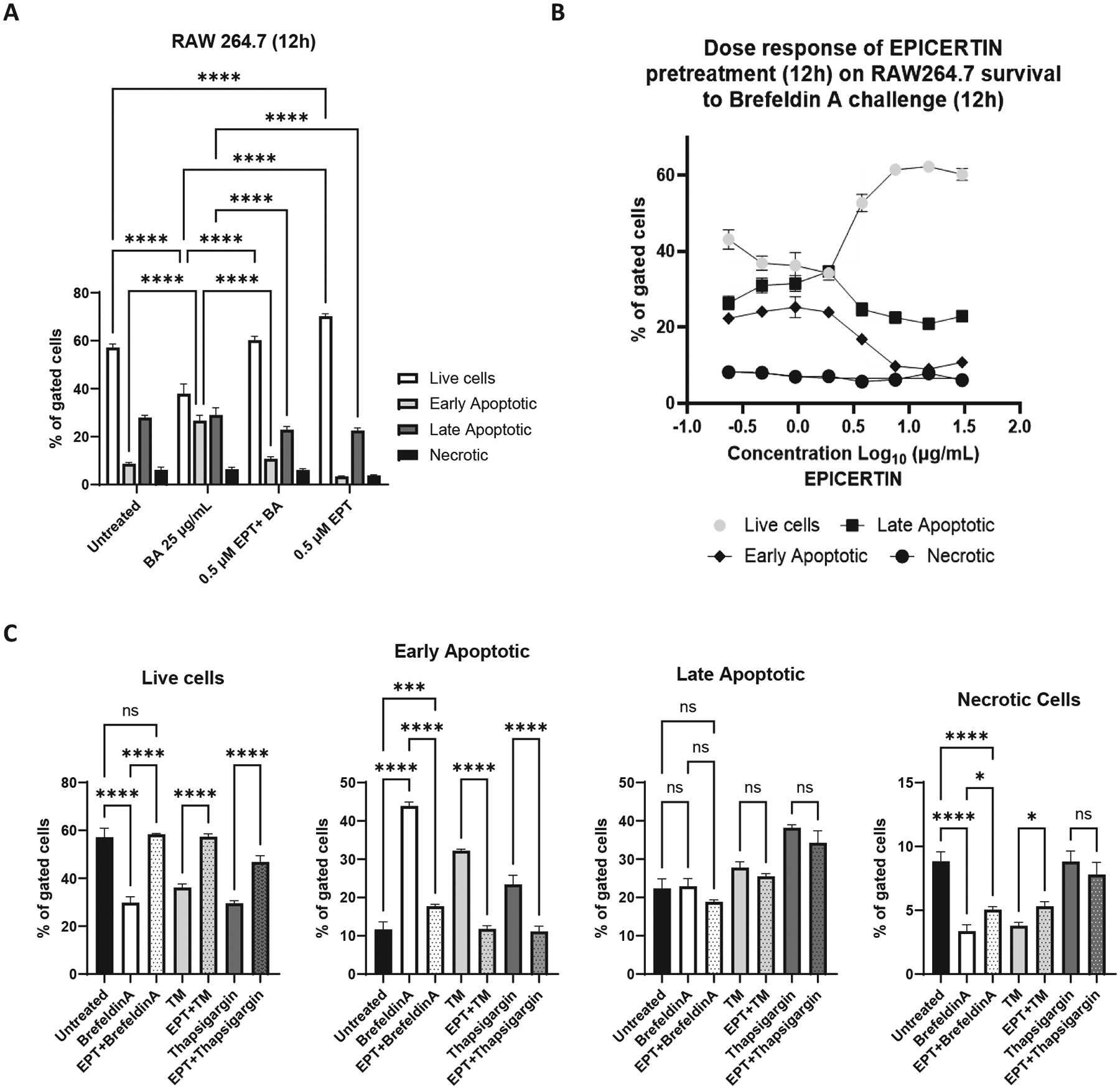
EPICERTIN prevents macrophage apoptosis induced by chemical ER-stress inducers. **A.** RAW264.7 macrophages pretreated for 12 h with 0.5 μM EPICERTIN significantly reduced the proportion of early and late apoptotic cells induced by brefeldin A (25 μg/mL). **B**. Dose-response effects of EPICERTIN on brefeldin A-induced ER stress and apoptosis. EPICERTIN in a dose dependent manner promoted an increase in viable cells while it reduced the proportions of early and late apoptotic cells induced by brefeldin A (25 μg/mL). The EPICERTIN EC50 values for promoting live cells and reducing early and late apoptotic cells were 3.67, 3.64, and 3.75 μg/mL, respectively, when treating 1 × 10^6^ cells. **C.** EPICERTIN promoted RAW264.7 cell viability and significantly reduced early apoptosis induced by brefeldin A (25 μg/mL), tunicamycin (5 μg/mL) and thapsigargin (5 μg/mL). One or two-way ANOVA with Bonferroni’s multiple comparison test was used to compare between groups. Significant differences are indicated with asterisks (*p < 0.05, **p < 0.01, ***p < 0.001, ****p < 0.0001). A representative experiment is shown in each panel, and each data point represents the mean ± SD of 5 (**A, B**) or 3 replicates per sample (**C**).

**Fig. 7.**
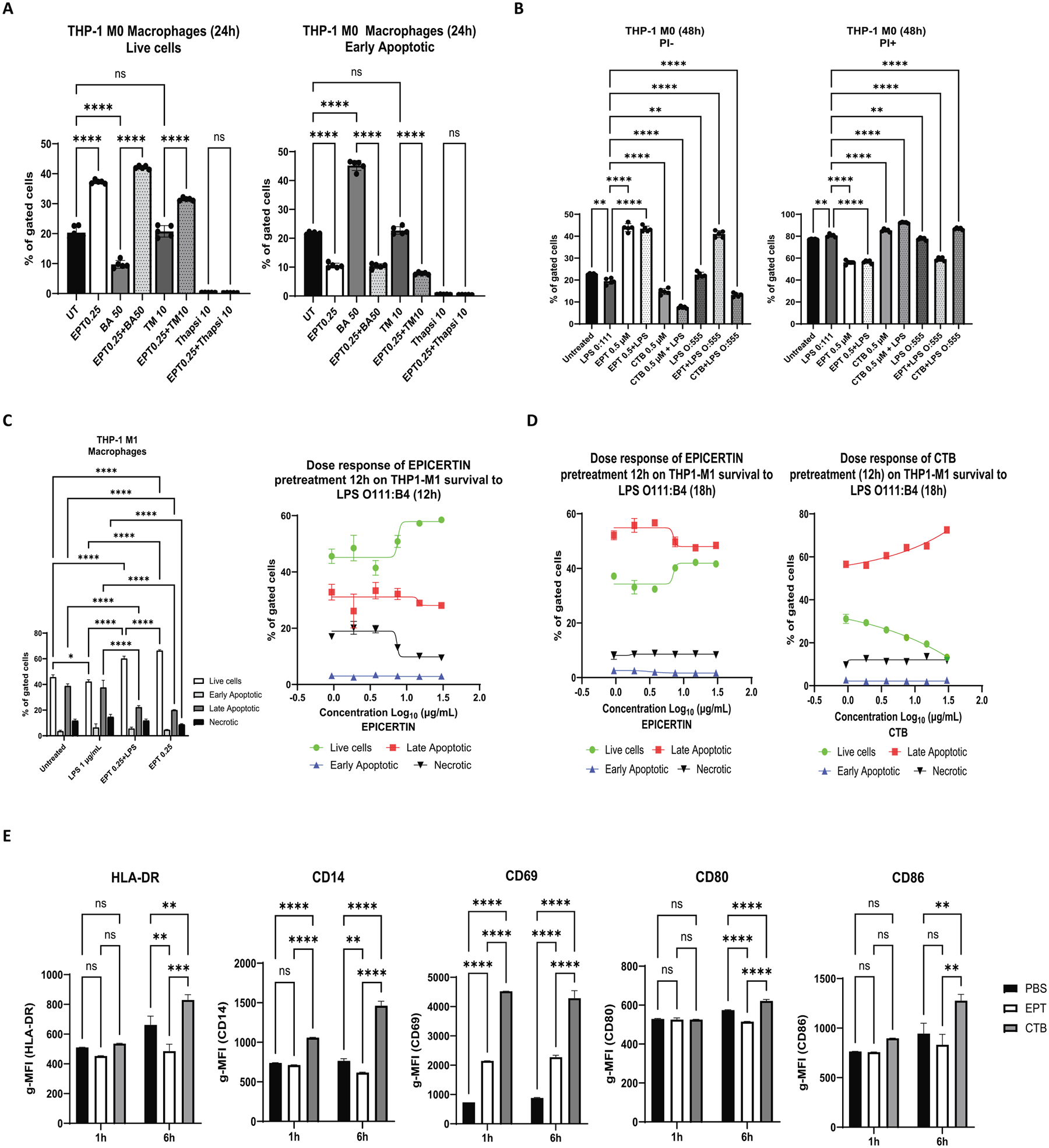
EPICERTIN prevents apoptosis of human macrophages through modulation of cell activation. **A**. EPICERTIN significantly (*p* < 0.0001) increased the proportion of live THP-1 M0 macrophages and blunted the apoptosis induced by brefeldin A. Tunicamycin did not have noticeable effects on apoptosis, however, EPICERTIN pretreatment increased viable cells and reduced early apoptotic cells that appear to occur by serum starvation. EPICERTIN pretreatment did not have a significant effect on thapsigargin-induced apoptosis. **B.** LPS O111:B4 modestly but significantly (*p* < 0.01) increased the proportion of total PI + THP-1 M0 macrophages at 48 h of treatment, whereas CTB induced a significant increase in apoptotic cell death (*p* < 0.0001), which was enhanced by the presence of LPS (O111:B4 or O:555). In contrast, EPICERTIN significantly promoted cell viability and reduced the proportion of dead cells regardless of the presence of LPS. **C**. EPICERTIN (0.25 μM) pretreatment of THP-1 M1 macrophages promoted survival and significantly reduced late apoptosis induced by LPS O111:B4. EPICERTIN in a dose-dependent manner significantly promoted cell viability and reduced the proportion of late apoptotic and necrotic cells induced by LPS O111:B4. The estimated EC50 value for EPICERTIN effects on live cells shown in this experiment was 7.1 μg/mL (1 × 10^6^ cells) with a range between 6.4 and 7.7 μg/mL between experiments. **D**. THP-1 M1 macrophages were pretreated with various doses of EPICERTIN or CTB for 12 h and LPSO111:B4 was added for an additional 18 h (total 30 h in serum-free conditions) before annexin V /PI staining. EPICERTIN still promoted cell viability and reduced late apoptotic cells induced by LPS, whereas CTB enhanced apoptosis. **E**. THP-1 M1 macrophages were cultured in complete RPMI medium in the presence of PBS, EPICERTIN (0.25 μM) or CTB (0.25 μM) and modulation of cell surface markers was detected after 1 and 6 h of culture by flow cytometry. CTB significantly increased the surface expression of HLA-DR, CD14, CD80, CD86 and CD69, while EPICERTIN significantly reduced the expression of those cell surface markers compared to PBS-treated cells and only mildly increased CD69 at levels significantly different from CTB or PBS. One or two-way ANOVA with Bonferroni’s multiple comparison test was used to compare between groups. Significant differences are indicated with asterisks (*p < 0.05, **p < 0.01, ***p < 0.001, ****p < 0.0001).

**Fig. 8.**
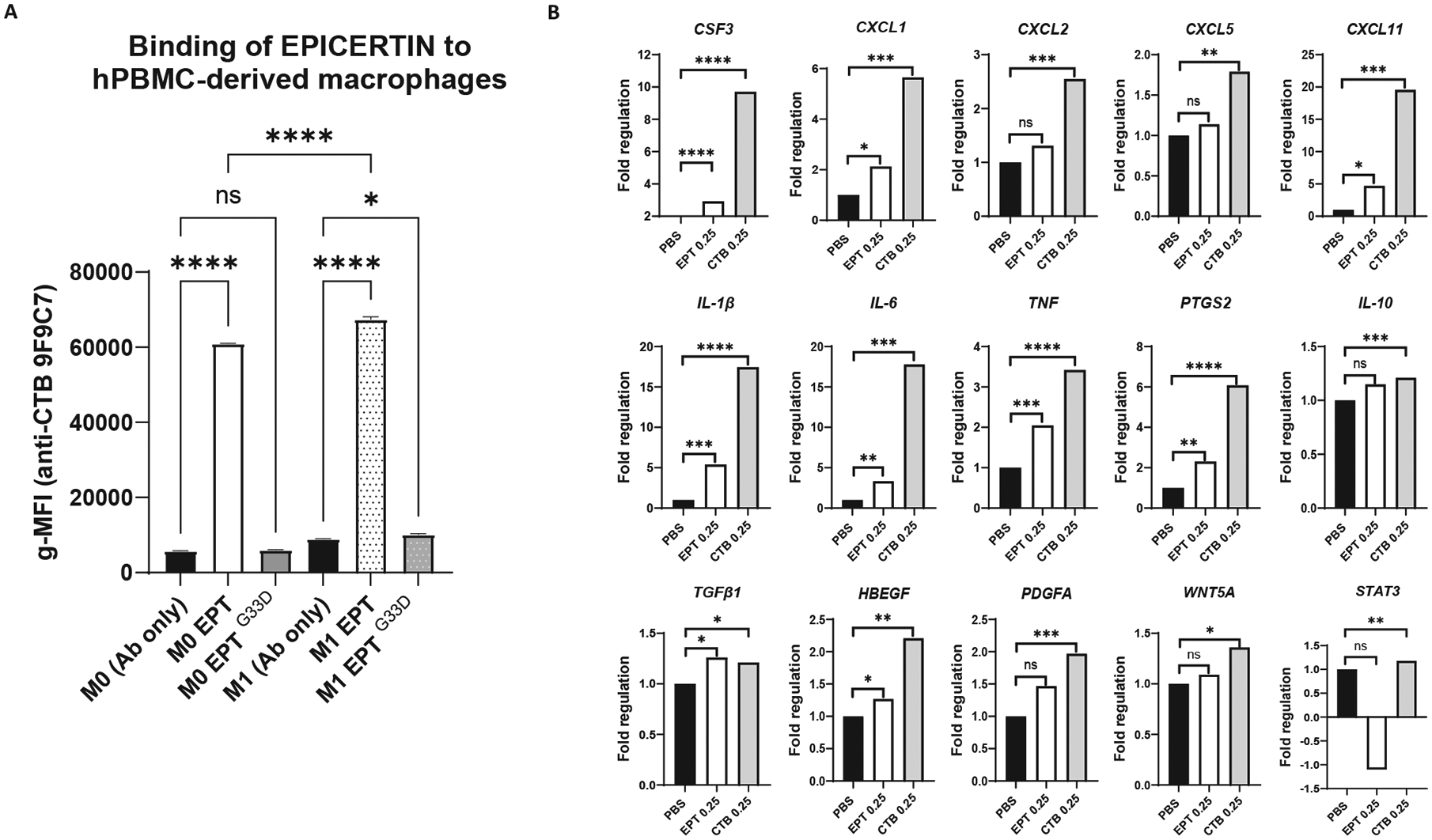
EPICERTIN modulates the activation of human PBMC-derived macrophages to maintain homeostasis. A. EPICERTIN binding to human PBMC-derived macrophages shows a significant (*p* < 0.0001) increase in binding upon M1 polarization with IFN-γ + LPS, whereas the mutant EPT^G33D^ showed no significant binding to the M0 macrophage stage, and it slightly increased to a significant level (*p* < 0.05) in the M1 polarized stage. B. EPICERTIN (0.25 μM) treated human PBMC-derived macrophages significantly reduced the transcription of proinflammatory cytokines and chemokines genes when compared to CTB, thus EPICERTIN favors homeostasis. In contrast, CTB induces a prominent proinflammatory response leading to cell death. One-way ANOVA with Bonferroni’s multiple comparison test was used to compare between groups (A). Significant differences are indicated with asterisks (*p < 0.05, **p < 0.01, ***p < 0.001, ****p < 0.0001). A single experiment is shown in each panel, and each data point represents the mean ± SD of 3 replicates. Gene expression in (B) is presented as Fold Regulation, calculated using the ΔΔCT method according to the Qiagen RT^2^ Profiler PCR array analysis workflow. For each gene, one ΔCT value was generated per biological replicate (n = 3 per treatment group), normalized to the panel’s designated housekeeping genes. Statistical significance was determined using an unpaired two-tailed *t*-test on the individual 2^−ΔCT^ values from the three biological replicates. Because Fold Regulation is a ratio derived from group-averaged 2^−ΔCT^ values (ΔΔCT method), standard deviation bars cannot be meaningfully applied and therefore not shown, consistent with manufacturer recommendations.

Here, we investigate the effects of EPICERTIN on macrophages and their potential contribution to the mucosal healing and anti-inflammatory effects in colitis. To address the role of EPICERTIN in macrophage regulation and mucosal healing, we evaluated its effects across multiple systems. These included a DSS-induced colitis model in mice (Figs. 1, 3A, B), murine RAW264.7 and peritoneal macrophages (Figs. 3C, D, 4–6), human THP-1-derived M0 and M1-like macrophages (Figs. 6–7), and human colon tissue from a patient with active colitis and PBMC-derived macrophages (Figs. 2, 8). Comparative studies with CTB were conducted in parallel to delineate the distinct effects of EPICERTIN in macrophage models. The data indicate that EPICERTIN promoted macrophage survival/homeostasis, whereas CTB induced apoptosis. EPICERTIN prevented macrophage apoptosis caused by chemical ER stress inducers or *E. coli* lipopolysaccharides (LPS). Finally, EPICERTIN modulated macrophage phenotype, and the transcription of proinflammatory cytokine and growth factor genes that impact diverse types of cells involved in colon tissue regeneration.

### Results

#### Oral treatment with EPICERTIN reduces neutrophil infiltration and enhances macrophage survival in the CLP of DSS colitis mice

Oral administration of 3 μg EPICERTIN, dosed twice (days 3 and 6) during DSS exposure, modulated various leukocyte populations in the CLP and mesenteric lymph nodes (MLNs) of DSS-colitis mice. These effects were observed two days post removal of DSS from the drinking water (Fig. 1), a time point when the healing effects begin to manifest as a reduced disease activity index (a composite measure of body weight, stool consistency, and bleeding).^21^ Specifically, EPICERTIN reduced neutrophil (Ly6G + ) infiltration and significantly increased both F4/80^+^CD11b^+^MHCII^hi^ (M1-like) and F4/80^+^CD11b^+^MHCII^lo/−^ (M2-like) macrophages in the CLP (Fig. 1B, C and Suppl. Fig. 1). The proportion of M2-like macrophages was superior to that of M1-like macrophages. Notably, the majority of M1-like (91.5%) and M2-like (62%) macrophages expressed CD206 and CX3CR1 receptors, and EPICERTIN binding to MHCII^hi^ was slightly higher than to MHCII^lo/−^ macrophages (Fig. 1C). In the MLNs, EPICERTIN also significantly increased dendritic cells (MHCII^hi^CD11b^−^CD103^+/−^), whereas no such increase was observed in the CLP. In addition, EPICERTIN tended to reduce both T and B lymphocytes in the CLP, while promoting a significant expansion of MHCII^+^CD19^+^ B lymphocytes in the MLNs (Suppl. Fig. 1D).

In a separate experiment, DSS colitis mice treated with lower doses of EPICERTIN (0.3 and 1.0 μg) exhibited dose-dependent modulation of wound healing-related gene expression, with significant upregulation of the colony stimulating factor 2 gene (*Csf2*), which is known to activate and promote macrophage survival and differentiation^26^, along with other cell growth factors, including *Hgf*, *Tgfa*, *Pdgfa*, and *Ffg10* (Fig. 1D). Transcripts associated with tissue repair, such as those encoding extracellular matrix components, remodeling enzymes, and integrins were also elevated (not shown). Notably, *Ctnnb1* and *Wnt5a*, key genes in epithelial regeneration pathways, were significantly upregulated (Fig. 1D), consistent with previous findings.^21^.

### EPICERTIN upregulates *CSF2* and various growth factor genes while suppressing proinflammatory mediators in colon tissue from a human with active colitis

Immunohistochemistry (IHC) analysis of human colon from a patient with active colitis revealed that EPICERTIN bound to the luminal side and basolateral membrane of colon crypt epithelial cells, and colocalized with HLA-DR^+^ leukocytes in the lamina propria (Fig. 2A). Furthermore, treatment of human colon tissue explants with EPICERTIN (1.0 μM) resulted in a time-dependent and significant upregulation of multiple cell growth factors genes, including *CSF2* (Fig. 2B), consistent with findings in the mouse colon (Fig. 1D). EPICERTIN also increased the expression of other cell growth factors that promote epithelial and stroma cell regeneration (*EGF*, *HGF*, *TGFB*, *PDGFA*, and *FGF10*) and tumor suppression (*PTEN1*), while repressing proinflammatory genes (*PTGS2*, *IL1B*, and *IL6ST*), among a broad array of wound healing genes that were up- and downregulated (Supplemental Table 1). These results suggest that, in addition to its previously demonstrated epithelial repair activity,^21^ EPICERTIN may promote mucosal healing by inducing an adaptive pro-survival response in macrophages, driven by the upregulation of cell growth factors that mediate tissue regeneration while concurrently suppressing inflammation.

#### Binding of EPICERTIN to macrophages is enhanced under inflammatory conditions

To investigate the effects of EPICERTIN on macrophages, we characterized the binding of EPICERTIN to murine primary CLP leukocytes under steady state and inflammatory conditions. EPICERTIN bound more strongly to F4/80^+^MHCII^hi^ macrophages than to F4/80^+^MHCII^lo/−^ macrophages in steady state, and this binding was significantly increased under DSS-induced inflammation (Fig. 3A). While EPICERTIN exhibited similar binding to F4/80^+^Ly6C high and low monocytes under steady state conditions, its binding to F4/80^+^ Ly6C^hi^ monocytes was reduced in the presence of inflammation (Fig. 3A). EPICERTIN also bound more strongly to MHCII^+^CD11c^hi^ dendritic cells than to MHCII^+^CD11c^lo^ counterparts in the presence of inflammation. In contrast, reduced binding of EPICERTIN was observed in CD8^+^ T cells and CD19^+^ B cells from the CLP in the presence of inflammation. A similar preferential binding of EPICERTIN to myeloid over lymphoid cells was observed in MLN leukocytes (Suppl. Fig. 2). However, EPICERTIN binding to inflamed MLN monocytes was less than that in healthy mice.

IHC of colon tissue isolated from a DSS colitis mouse following oral administration of EPICERTIN revealed that the protein localized within the cytoplasm of colon crypt epithelial cells, and it was also taken up by MHCII^+^F4/80^+^ macrophages in the CLP. These macrophages were primarily located just underneath the basement membrane at the base of colonic crypts, with additional EPICERTIN-positive macrophages observed around the mid-region of the crypt (Fig. 3B).

Taken together, these results suggest that EPICERTIN preferentially binds to MHCII^+^ macrophages in the CLP rather than to lymphocytes under steady-state conditions, and that this binding is further enhanced upon immune activation or inflammation.

To further explore EPICERTIN’s interaction with macrophages, we used the RAW264.7 murine macrophage cell line. Flow cytometry analysis of geometric mean fluorescence intensity (gMFI) revealed that EPICERTIN and CTB exhibited nearly identical binding to RAW264.7 cells under steady-state conditions. However, binding was significantly increased upon co-incubation with the Toll-like receptor 4 (TLR4) agonist LPS R515 and was significantly reduced upon co-incubation with the macrophage differentiation inducer PMA (Fig. 3C). Western blot analysis further confirmed that treatment of RAW264.7 with EPICERTIN and CTB (0.25 μM) resulted in similar amounts of protein bound to or internalized by the macrophages over a 24 h culture period in complete DMEM medium (Fig. 3D). There was, however, a trend of increased amounts of EPICERTIN at 12 h and 24 h compared to CTB when normalized to β-actin (Fig. 3D), potentially reflecting prolonged ER retention of EPICERTIN via KDELR interaction, as demonstrated previously.^21^.

#### EPICERTIN promotes survival whereas CTB induces apoptosis in RAW264.7 macrophages

We next analyzed the effects of EPICERTIN and CTB binding on macrophages. RAW264.7 cells, treated with EPICERTIN (0.03–0.5 μM) for 12 h under serum-free conditions, showed an average of 60.3% viable cells, while the proportion of late apoptotic cells ranged from 22.2% to 29% with no clear dose-dependent trend (Fig. 4A). In comparison, PBS or *E. coli* R515 LPS (1 μg/mL) showed 25% and 29% of late apoptotic cells, respectively. In contrast, CTB significantly reduced viable cells and markedly increased the proportion of late apoptotic cells in a dose-dependent manner compared to PBS control, with late apoptotic cells increasing from 26% at the lowest dose to 73% at the highest dose (Fig. 4A). Further experiments were conducted over a 48-h period under serum-free conditions. By 48 h, LPS significantly reduced the proportion of live cells and increased necrotic cells compared to the PBS control (Fig. 4B). EPICERTIN significantly promoted cell survival and significantly reduced late apoptotic cells compared to PBS, LPS R515 or CTB groups. Conversely, CTB significantly reduced live cells and increased both early and late apoptotic cells or total PI^+^ cells (Fig. 4B). A similar pattern of reduced cell viability and increased late apoptotic and necrotic cells was observed with a commercial (Sigma Aldrich) recombinant CTB (crCTB), produced in a eukaryotic cell line (HEK293). This protein, which had comparable low levels of LPS contamination (<0.6 EU/mg) to our in-house CTB, induced apoptosis in RAW264.7 macrophages at both high and low doses tested at 6 or 12 h, respectively, under serum-free conditions (Suppl. Fig. 3A, B). These findings suggest that the apoptotic effects of CTB on RAW264.7 cells are intrinsic to the protein and independent of the production method.

### EPICERTIN promotes the survival of resident peritoneal macrophages

Resident peritoneal macrophages from healthy C57BL/6 mice were treated with EPICERTIN or CTB (0.125 or 0.5 μM) under serum-free conditions for 12 h to assess cell viability. *E. coli* LPS R515, used as reference control, significantly reduced live cells. EPICERTIN maintained cell viability and showed a trend toward increased live cells and decreased late apoptotic cells, although these effects were not statistically significant. In contrast, CTB significantly reduced live cells and increased both late apoptotic and total PI^+^ macrophages compared to both PBS and EPICERTIN, particularly at the higher dose (Fig. 5). A similar reduction in live cells and increase in late apoptosis was also observed with crCTB compared to EPICERTIN or PBS control (Suppl. Fig. 4). Interestingly, despite its lack of a significant impact on live cells, EPICERTIN significantly increased early apoptotic cells compared to PBS. However, the proportion of early apoptotic cells remained much lower than that of live and late apoptotic cells (Suppl. Fig. 4).

Immunocytochemistry of peritoneal macrophages treated with EPICERTIN showed relatively smooth, rounded cell surfaces, and some cells maintained a fusiform, adherent morphology. In contrast, CTB-treated macrophages showed irregular cell surfaces with swollen and vacuolated cytoplasm, indicative of major alterations in cell physiology (Fig. 5B).

Apoptosis of resident peritoneal macrophages was confirmed by the terminal deoxynucleotidyl transferase dUTP nick-end labeling (TUNEL), which detects DNA fragmentation. The number of TUNEL^+^ peritoneal macrophages treated with EPICERTIN was not significantly different from the PBS-treated controls. In contrast, CTB significantly increased TUNEL^+^ peritoneal macrophages after 3 h of culture compared to PBS (Fig. 5C).

To further assess the effects of EPICERTIN and CTB on resident peritoneal macrophages, cells were treated with EPICERTIN or CTB (0.5 and 0.125 μM) for 24 h, and the levels of cell surface markers were analyzed by flow cytometry. Comparison of gMFI indicated that EPICERTIN significantly reduced MHCII and F4/80 expression, whereas CTB showed opposed effects, significantly increasing MHCII and F4/80 levels, particularly at the higher concentration tested (Suppl. Fig. 5). These findings indicate that EPICERTIN promotes the survival of resident peritoneal macrophages, which is associated with reduced expression of cell surface molecules. In contrast, CTB induces increased expression of surface MHCII and F4/80 and pronounced morphological changes and DNA fragmentation, which are characteristics of apoptosis or activation-induced cell death.

### Pro-survival effects of EPICERTIN prevent apoptosis of RAW264.7 macrophages induced by chemical ER-stress inducers

Excessive inflammation, ER stress and aberrant UPR activation in colon epithelial cells have been linked to the pathogenesis of human colitis.^5,27,28^ We investigated whether EPICERTIN could prevent or delay macrophage apoptosis induced by standard chemical ER stress inducers. Treating RAW264.7 cells with the ER stressor brefeldin A for 12 h in serum-free medium significantly reduced the proportion of live cells and significantly increased early apoptotic cells, with no significant changes in late apoptotic or necrotic populations (Fig. 6A). However, pretreatment of these cells with EPICERTIN (0.5 μM) for 12 h prior to brefeldin A challenge significantly reversed the reduction in live cell numbers and decreased the proportion of early apoptotic cells induced by brefeldin A (Fig. 6A); these protective effects of EPICERTIN were dose dependent (Fig. 6B). The EC50 value of EPICERTIN to reduce early apoptotic cells was 3.75 μg/mL, while its EC50 value for promoting live cells was 3.67 μg/mL. In addition to enhancing cell survival and reducing early apoptosis, EPICERTIN also decreased the proportion of late apoptotic cells (Fig. 6A, B). In contrast, CTB significantly reduced the proportion of live cells and increased late apoptotic cells in a dose-dependent manner upon brefeldin A challenge (Suppl. Fig. 6). Moreover, EPICERTIN (0.25 μM) almost completely reversed the reduction in live cell numbers and markedly reduced the proportion of early apoptotic cells induced by two additional, commonly used ER stress inducers, tunicamycin and thapsigargin (Fig. 6C). Collectively, these results demonstrate that, in stark contrast to CTB, EPICERTIN induces an adaptive pro-survival response that protects RAW264.7 macrophages from apoptosis triggered by various ER stressors, including inhibitors of vesicular trafficking (brefeldin A), protein glycosylation (tunicamycin), and ER Ca^2+^ ATPase (thapsigargin).

### EPICERTIN modulates the activation of human macrophages and prevents apoptosis induced by chemical ER stressors or LPS

We next investigated whether the pro-survival effects of EPICERTIN observed in murine macrophages could be translated to human macrophages. EPICERTIN showed strong binding to the human THP-1 monocyte cell line upon activation with PMA (M0 differentiation), and this binding was further increased upon M1 polarization with IFNγ + LPS (Suppl. Fig. 7A). This interaction was mediated through GM1, as EPICERTIN^G33D^, a variant lacking GM1 binding affinity, failed to exhibit such prominent binding to either THP-1 or PBMC-derived macrophages (Suppl. Fig. 7A **and** Fig. 8A). Culturing THP-1 M0 macrophages in serum-free medium for 24 h significantly reduced cell viability to approximately 20%. However, treatment with EPICERTIN (0.25 μM) nearly doubled the number of live cells and significantly reduced the proportion of early apoptotic cells (Fig. 7A). Exposure to brefeldin A (50 μg/mL) or thapsigargin (10 μg/mL) further exacerbated cell death and increased early apoptosis, whereas tunicamycin (10 μg/mL) did not induce additional cytotoxicity beyond that caused by serum-free conditions. Pretreatment with EPICERTIN significantly reduced the proportion of early apoptotic cells and increased the proportion of live cells in the presence of brefeldin A and tunicamycin. However, EPICERTIN provided no protective effect against thapsigargin-induced cell death, which resulted in nearly complete loss of cell viability (Fig. 7A).

EPICERTIN’s protective effects against LPS challenge were also evaluated in THP-1 M0 macrophages. PI staining revealed that treatment with LPS O111:B4 (1 μg/mL) induced a marginal but statistically significant further reduction of live cells and an increase in PI^+^ cells, beyond the effects of serum-free culture alone. EPICERTIN treatment significantly improved cell viability, doubling the proportion of live cells and reducing the proportion of dead cells, regardless of the presence or absence of LPS O111:B4 or LPS O55:B5 (Fig. 7B). In contrast, CTB markedly reduced the proportion of live cells and increased dead cells, and these effects were even more pronounced in the presence of LPS O111:B4 and O55:B5 (Fig. 7B).

We next examined M1-polarized macrophages. While 10 μg/mL tunicamycin did not significantly affect the proportions of live (PI^−^) or dead (PI^+^) cells beyond the viability reduction caused by serum-free culture, EPICERTIN again demonstrated a protective effect by increasing the viability of M1-polarized THP-1 both in the presence and absence of tunicamycin, whereas CTB further reduced the proportion of live cells (Suppl. Fig. 7D). LPS O111:B4 induced a marginal but statistically significant reduction in the proportion of live cells compared to untreated M1 macrophages (Fig. 7C). Pretreatment with EPICERTIN significantly increased live cells and reduced late apoptotic and necrotic M1 macrophages, both in the presence and absence of LPS O111:B4. Notably, the protective effect of EPICERTIN in the presence of LPS was dose dependent. The best-fit EC50 value for EPICERTIN in promoting the viability of THP-1 M1 macrophages was 7.1 μg/mL (per 1 × 10^6^ cells) and ranged from 6.4 to 7.7 μg/mL across experiments. We further evaluated the protective effect of EPICERTIN in THP-1 M1 macrophages by extending the duration of LPS exposure by an additional 18 h. At the time of analysis (total 30 h in serum-free conditions), EPICERTIN promoted macrophage survival in a dose-dependent manner, with the best-fit EC50 values of 7.06 and 7.12 μg/mL (per 1 × 10^6^ cells) for promoting live cells and reducing late apoptotic cells, respectively. On the other hand, CTB decreased live cells while increasing late apoptotic cells, with EC50 values of 7.78 and 7.05 μg/mL, respectively (Fig. 7D).

We also investigated the effects of EPICERTIN (0.25 μM) or CTB (0.25 μM) on the modulation of cell surface markers in THP-1 M1 macrophages using flow cytometry. Analysis of gMFI revealed that EPICERTIN increased the surface expression of CD69 as early as 1 h post-treatment and reduced the surface levels of HLA-DR, CD14, and CD80 by 6 h. In contrast, CTB induced a significant increase in the expression of HLA-DR, CD14, CD80, CD86 and CD69 (Fig. 7E). Notably, the increase of CD69 was more pronounced with CTB than with EPICERTIN, and CD14 upregulation was already significant at 1 h following CTB treatment.

Taken together, these findings indicate that macrophage activation enhances the binding of EPICERTIN and CTB. However, this binding leads to distinct phenotypic outcomes: EPICERTIN promotes a less inflammatory macrophage phenotype, whereas CTB appears to induce a proinflammatory state that may lead to activation-induced cell death. Remarkably, EPICERTIN promoted survival and prevented apoptosis of human THP-1 macrophages, even in the presence of LPS, while CTB consistently promoted apoptosis.

### EPICERTIN and CTB differentially modulate the expression of inflammatory mediator genes in human PBMC-derived macrophages

Finally, we investigated the effects of EPICERTIN and CTB (0.25 μM) on the expression of genes associated with inflammatory pathways in human PBMC-derived macrophages. Consistent with observations in THP-1 cells, increased binding of EPICERTIN to human PBMC-derived macrophages was confirmed upon activation or M1 polarization (Fig. 8A). Next, we used a human wound healing RT^2^ Profiler PCR array to assess the expression of 84 key genes in wound healing. A clustergram of the response showed that EPICERTIN clustered closer to the PBS control than CTB (Suppl. Fig. 8). Notably, CTB induced a pronounced upregulation of chemokine and proinflammatory cytokine genes compared to PBS-treated controls, except for *IL10* and *TGFB1*, which showed only modest increases. Among the most highly upregulated genes following CTB treatment were *CXCL11*, *IL1B*, and *IL6*, each showing over a 15-fold increase relative to PBS controls. In contrast, EPICERTIN induced only marginal upregulation of these genes (Fig. 8B). These findings are consistent with the surface marker analysis of THP-1 cells (Fig. 7E), in which CTB promoted a proinflammatory macrophage phenotype, whereas EPICERTIN favored a less inflammatory phenotype and cell homeostasis.

### Discussion

EPICERTIN, an engineered variant of the cholera vaccine antigen CTB containing an Asn4 → Ser substitution and a C-terminal ER retention motif, has shown promise as an oral biotherapeutic for promoting mucosal healing. Previous studies have demonstrated that EPICERTIN facilitates epithelial restitution and suppresses inflammation in both murine DSS-induced colitis models and human IBD colonic explants.^19,21,22,29,30^ In these prior studies, EPICERTIN was also referred to as CTB-KDEL^21^ or CTBp^22^; these terms describe the same engineered CTB variant containing a C-terminal ER-retention signal and differ only in nomenclature or production context. However, the underlying mechanism driving its anti-inflammatory effects remains poorly understood. The present study aimed to address this knowledge gap by investigating the immunomodulatory effects of EPICERTIN on macrophages, key players in intestinal inflammation and tissue homeostasis.

The present study demonstrated that oral administration of EPICERTIN in DSS-colitis mice significantly reduced neutrophil infiltration and preferentially recruited M2-like over M1-like macrophages into the CLP (Fig. 1). Additionally, EPICERTIN significantly upregulated *CSF2* along with other genes involved in tissue repair, while downregulating proinflammatory genes in colon tissue from a patient with active colitis (Fig. 2B). These findings are similar to our previous observations^21,22^ and further support the mucosal healing effect of EPICERTIN. M2 macrophages are key mediators of wound healing and tissue remodeling, while CX3CR1^+^ macrophages are essential for maintaining gut integrity, resisting microbial invasion, and promoting a tolerogenic gut immune environment to mucosa-associated commensal bacteria.^31 9,32^ We acknowledge that the classification of macrophages as M1 or M2 in this study was based on the expression of MHCII (hi and low/-), CD206, and CX3CR1 surface receptors. Therefore, the terms “M1-like” and “M2-like” were used to reflect phenotypic resemblance rather than definitive functional polarization. We also acknowledge that the gated population may include a minor contribution from CD103^−^CD11b^+^ dendritic cells with low to intermediate F4/80 and CX3CR1 expression, as previously described in the CLP under inflammatory conditions.^33^.

Our results indicate that EPICERTIN favors the differentiation or recruitment of M2-like over M1-like macrophages, possibly through modulation of cell surface molecule expression and function. In contrast to CTB and the *E. coli* heat-labile enterotoxin B subunit (LTB), both of which have been shown to upregulate the expression of MHCII and costimulatory receptors,^34–37^ EPICERTIN reduced their expression on both mouse and human macrophages (Suppl. Fig. 5, Fig. 7E) and only mildly increased the CD69 activation marker (Fig. 7E). EPICERTIN binding to MHCII^hi^ macrophages in the CLP appears to modulate their phenotype, promoting a less inflammatory phagocytic profile that may be protective against the progression of IBD.^7^ Further studies are warranted to investigate the potential of EPICERTIN to induce macrophage dedifferentiation, phenotype switching, or reprogramming.

EPICERTIN and CTB both bind to GM1 ganglioside receptors located within lipid rafts of the plasma membrane, mediating their internalization and retrograde transport to the ER.^20^ The present study demonstrated that the binding of EPICERTIN or CTB to macrophages is enhanced upon activation, polarization, or LPS stimulation – conditions that mimic the inflammatory environment observed in colitis (Fig. 3 **and** Suppl. Fig. 7). LPS, a TLR4 ligand, is elevated in the inflamed colonic mucosa and in the plasma of IBD patients as a consequence of intestinal barrier dysfunction.^38^ Myeloid cells from IBD patients show increased expression of TLR4, greater LPS uptake, and significantly higher secretion of pro-inflammatory cytokines compared to cells from non-IBD controls.^39^ Meanwhile, recent studies have highlighted a significant contribution of ER stress in IBD pathogenesis^5,40^, including the modulation of macrophage functions in the colon.^41^ Notably, EPICERTIN promoted the survival of both mouse and human macrophages, whereas CTB significantly reduced macrophage viability (Figs. 4, 5). EPICERTIN promoted the survival of mouse RAW264.7 and human THP-1 macrophages with estimated EC50 values in the mid-nanomolar range (50 nanomolar corresponding to approximately 3 μg/mL), whereas CTB at those levels induced cell death, likely through apoptosis or pyroptosis, as annexin V binding does not discriminate these forms of cell death (Figs. 4–7).^42^ Furthermore, the prosurvival effects of EPICERTIN overcame the cytotoxic impacts of chemical ER stressors and LPS (Figs. 6, 7), supporting its clinical relevance and potential therapeutic benefits for treating IBD.

However, the underlying mechanisms responsible for the opposite effects of EPICERTIN and CTB on macrophage viability and phenotype remain unclear. CTB has previously been shown to induce the apoptosis of splenic CD8^+^ T cells isolated from MLNs,^35^ and its close relative, *E. coli* LTB, was also reported to induce apoptosis of CD8^+^ T cells via NF-κB and caspase-3 activation.^43^ To our knowledge, the present study is the first to demonstrate that CTB can induce apoptosis in macrophages under serum-free conditions. Kaisho and colleagues previously showed that CTB synergized with LPS O111:B4 to induce IL-1β production in both bone marrow-derived and resident peritoneal macrophages, through activation of pyrin and/or NLRP3 inflammasomes involving the ER stress sensor IRE1α-XBP1 pathway.^44,45^ Synergistic activation of IRE1α-XBP1 axis, through both LPS-TLR4 pathway and the ER stress-induced UPR, is necessary for maximum cytokine production in macrophages^46^ and, likewise, in dendritic cells.^47^ This activation is accompanied by increased glycolysis, poly ADP-ribose polymerase and NADPH oxidase activation, NAD + imbalance and reactive oxygen species (ROS) mediated DNA damage.^48^ Consistent with these findings, our gene expression analysis of human PBMC-derived macrophages revealed that CTB markedly enhanced *IL1B* expression (>15-fold), along with upregulation of other chemokine and proinflammatory cytokine genes, whereas EPICERTIN induced only a moderate increase (<5-fold). Dysregulated production of proinflammatory cytokines, including IL-1β, Il-6, and TNF-α, is known to promote apoptotic cell death.^46,49,48^ Moreover, CTB-induced macrophage cell death was further exacerbated in the presence of LPS O111:B4, whereas EPICERTIN protected against LPS-induced cytotoxicity in both RAW264.7 and THP-1 cells (Suppl. Fig. 3C, Fig. 7). Thus, the differences in macrophage survival between CTB and EPICERTIN may be partly explained by their differential interaction with LPS, either through direct binding^50^ or modulation of downstream signaling pathways. However, this does not fully account for the protective effects of EPICERTIN or the contrasting responses observed between EPICERTIN and CTB in the presence of chemical ER stress inducers (Figs. 6, 7). Further studies will be necessary to elucidate these mechanisms.

To further investigate the contrasting effects of EPICERTIN and CTB on macrophage biology, we performed RNA-seq analysis on human PBMC-derived M0 macrophages treated for 12 h with EPICERTIN (0.25 μM), CTB (0.25 μM), or PBS (Suppl. Fig. 9A–D). Both EPICERTIN and CTB elicited large, overlapping transcriptional responses, yet the magnitude and directionality of these changes differed substantially, resulting in clear separation in principal component analysis (Suppl. Fig. 9A, B). Consistent with our qPCR findings (Fig. 8B), CTB induced a robust proinflammatory program, whereas EPICERTIN activated similar gene sets but with markedly attenuated amplitude and concurrent induction of regulatory mediators. For example, *IL1B* and *IL6* were upregulated by both proteins relative to PBS but to a lesser extent with EPICERTIN (*IL1B*: log_2_FC +2.46 for EPICERTIN vs. +3.87 for CTB; *IL6*: +2.35 vs. +4.07, respectively). This pattern extended to *TNF*, *CXCL9*, and *CXCL10*, each showing more moderate induction by EPICERTIN compared with CTB (Suppl. Table 2). In addition to cytokine differences, EPICERTIN and CTB diverged in their regulation of NF-κB-associated transcriptional pathways. CTB strongly upregulated both *NFKB1* (log_2_FC +0.89) and *NFKB2* (log_2_FC +1.02), whereas EPICERTIN induced only modest increases (*NFKB1* +0.27; *NFKB2* +0.45) relative to PBS (Suppl. Table 2). These findings indicate that CTB drives substantially stronger NF-κB activation, while EPICERTIN engages a more tempered inflammatory transcriptional profile.

In parallel, EPICERTIN upregulated several survival-associated genes (e.g., *DAD1*^51^) and enriched transcription factors linked to stress tolerance and metabolic adaptation, including *ATF4* (integrated stress response)^52^, *JUND* (oxidative/metabolic stress adaptation)^53^, and *NFE2L3* (ER stress and antioxidant signaling)^54^, each showing modest induction with EPICERTIN but little or no induction with CTB (Suppl. Table 2). Notably, EPICERTIN uniquely upregulated KDEL receptor genes (*KDELR1*, log_2_FC +0.26; *KDELR2*, +0.28), consistent with its engineered C-terminal KDEL motif and with prior findings that this motif can engage adaptive UPR programs in epithelial systems^21^, whereas CTB showed no such induction (Suppl. Table 2). EPICERTIN further increased expression of the UPR-associated transcription factor *DDIT3*/*CHOP* (log_2_FC +0.57), whereas CTB downregulated it (log_2_FC −0.33), supporting the idea that EPICERTIN triggers a controlled, adaptive ER-stress response rather than a terminal stress program.

Collectively, these findings indicate that EPICERTIN tempers the intensity of macrophage inflammatory activation while simultaneously promoting pro-survival and stress-adaptive pathways, whereas CTB drives stronger proinflammatory signaling and a transcriptional profile more consistent with stress susceptibility. Future studies examining cytokine secretion and signaling at the protein level will be important to validate these transcriptional signatures and further define EPICERTIN’s anti-inflammatory mechanisms in macrophages.

A dysregulated UPR signaling due to prolonged ER stress has been implicated in the pathogenesis of IBD.^23,27,28^ It is unclear, however, whether UPR dysfunction is a cause or a consequence of the excessive cell death and inflammation seen in IBD patients. We previously showed that EPICERTIN induces an adaptive UPR in colon epithelial cells, accompanied by TGFβ1 secretion, and promotes a regenerative response in human IBD colon tissue,^21^ suggesting that EPICERTIN-induced adaptive UPR may confer therapeutic benefit in IBD. In this study, EPICERTIN modestly but significantly increased the transcription of various growth factor genes, including *CSF2, EGF* and *TGFB1* in human colon tissue with active colitis (Fig. 2), and also upregulated *TGFB1* expression in human PBMC-derived macrophages (Fig. 8). Monocytes and macrophages are known as the primary sources of TGFβ in irradiation-induced colitis, where this cytokine induces a fetal-like regenerative state in the epithelium via activation of pro-regenerative YAP and SOX9 transcription factors.^23^ We also found that EPICERTIN-treated human PBMC-derived macrophages significantly increased heparin-binding epithelial-like growth factor (*HBEGF*) transcription, at levels higher than *TFGB1* (Fig. 8B). Notably, HBEGF is known to be produced by type 3 innate lymphoid cells and has a protective effect on the intestinal epithelium against TNF-induced cell death.^55^.

Collectively, we propose that, as observed in colon epithelial cells,^21^ EPICERTIN modulates the activation status of macrophages through mild ER stress and adaptive UPR response induction. This may regulate the production of proinflammatory mediators that attract neutrophils, while promoting the secretion of TGFβ and other growth factors that support the survival of macrophages, epithelial cells, and stroma cells in response to environmental insults and ER stressors present in the inflamed colon. Thus, EPICERTIN promotes a less inflammatory, pro-remodeling macrophage phenotype that contributes to re-establishing epithelial barrier integrity in colitis. Future studies dissecting the specific UPR pathways engaged by EPICERTIN, particularly the contribution of IRE1α, PERK and ATF6 sensors in macrophages and how these responses differ from those elicited by CTB, will be important to define its mechanistic basis for mucosal healing. In addition, cell type-specific approaches such as conditional macrophage depletion or reporter models will be essential to delineate the precise contribution of macrophages to EPICERTIN’s protective effects *in vivo*.

### Conclusion

We evaluated the role of EPICERTIN in macrophage regulation and mucosal healing across multiple systems, including a murine DSS-induced colitis model, murine RAW264.7 and peritoneal macrophages, human THP-1-derived M0 and M1-like macrophages, human colon tissue from a patient with active colitis, and human PBMC-derived macrophages. Our results demonstrated that EPICERTIN promotes macrophage survival and protects against apoptosis induced by LPS and ER stress. It preferentially interacts with activated macrophages and downregulates the expression of surface activation markers, thereby supporting a less inflammatory, pro-remodeling macrophage phenotype. In contrast, CTB exerts opposing effects, markedly upregulating chemokine and proinflammatory cytokine gene expression and promoting activation-induced cell death. These findings provide new insight into the therapeutic potential of EPICERTIN in colitis, highlighting its ability to promote mucosal healing and tissue regeneration.

### Methods

#### Animal care

All experiments using C57BL/6J mice were performed at the University of Louisville and approved by the Institutional Animal Care and Use Committee. General procedures for animal care and housing followed the current Association for Assessment and Accreditation of Laboratory Animal Care recommendations, current requirements stated in the Guide for the Care and Use of Laboratory Animals (National Research Council), and current requirements as stated by the U.S. Department of Agriculture through the Animal Welfare Act and Animal Welfare regulations (July 2020).

### Production of EPICERTIN and CTB in *Escherichia coli*

EPICERTIN and the parental molecule CTB (with a C-terminal six-histidine tag) expression vectors, pNM569 and pNM036, respectively, encoded in the pET-22b(+) backbone, were used to transform BL21 (DE3) *E. coli* competent cells. Bacterial cultures were induced with 0.4 mM IPTG for 4 h and the secreted recombinant protein was purified by Talon Superflow Metal (Cobalt) affinity chromatography resin using an ÄKTA purification system (GE healthcare), as previously described.^18^ A non-GM1-binding mutant EPICERTIN (EPT^G33D^) containing an aspartic acid substitution for glycine at position 33 (G33D) produced in *Nicotiana benthamiana* was described previously.^21^ Endotoxin levels in CTB and EPICERTIN preparations were < 1 EU/mg of protein, as determined by the Charles River PTS Endotoxin test system (Charles River).

### Dextran sulfate sodium-induced colitis and EPICERTIN administration to mice

DSS-induced colitis was established in 8 to 12-week-old female C57BL/6J mice obtained from Jackson Laboratories (Bar Harbor, ME), by administering 3% DSS (36,000–50,000 MW; MP Biomedical) in drinking water for seven days as previously described^21^. EPICERTIN (3, 6 or 10 μg) or 100 μL of PBS were administered by oral gavage using oral gavage needles. Oral administration of test compounds was performed immediately after neutralization of the gastric acid with 200 μL of sodium bicarbonate (30 mg/mL). Colon tissues were collected at indicated time points for immune cell isolation or embedded in OCT compound for cryosectioning and immunohistochemistry.

### Cell culture and cell viability/apoptosis assay

Primary peritoneal macrophages from healthy C57BL/6J mice and RAW264.7 macrophages (ATCC, TIB-71) were grown in complete DMEM medium (supplemented with 10% FBS, P/S, HEPES, and sodium pyruvate). Macrophages (1 × 10^6^ cells/ ml) were cultured in 5 mL polystyrene tubes and treated with PBS, LPS, CTB, or EPICERTIN at the indicated concentrations in serum-free DMEM medium containing 0.1% BSA (Sigma Aldrich) for the indicated time points before Annexin V/propidium iodide (PI) staining. Annexin V and PI were used to discriminate live, early apoptotic, late apoptotic, and necrotic cells. Early apoptotic cells expose phosphatidylserine and stain Annexin V-positive while retaining membrane integrity and excluding PI, whereas late apoptotic cells lose membrane integrity and therefore stain positive for both Annexin V and PI. Cells were pretreated with EPICERTIN or CTB for 12 h in serum-free DMEM medium containing 0.1% BSA. Brefeldin A (10–25 μg/mL),^56^ tunicamycin (5 μg/mL),^57^ or thapsigargin (5 μg/mL) was added and cells were analyzed 12 h later. Human THP-1 monocytes (ATCC,TIB-202) were cultured in complete RPMI 1640 and differentiated into M0 macrophages by adding 100 ng/mL phorbol 12-myristate 13-acetate^58^ for 48 h and rested in complete RPMI medium for 24 h before treatment. THP-1 M0 macrophages were polarized into M1 macrophages by adding 50 ng/mL recombinant human IFN-γ and 15 ng/mL of *E. coli* LPS R515 for 48 h and rested in complete RPMI medium for 24 h before treatment.^59^ Human macrophages were cultured in 5 mL polystyrene tubes, pretreated with varying concentrations of EPICERTIN or CTB for 12 h in serum-free RPMI medium containing 0.1% BSA, and subsequently challenged with brefeldin A (25–50 μg/mL), tunicamycin (10 μg/mL), thapsigargin (10 μg/mL)^60,61^ or LPS for additional 12 or 18 h before Annexin V/PI staining and flow cytometry analysis. Human PBMC-derived macrophages were generated from human PBMC treated with recombinant human M–CSF (CSF1, 50 ng/mL) in complete RPMI for 6 days in 6-well plates and subsequently treated with test compounds for 12 h in RPMI medium with 2% FBS, after which total RNA was isolated for qRT-PCR analysis.

### Human colon tissue culture

All research involving human tissues followed relevant guidelines and regulations established by the University of Louisville Institutional Review Board Committee. Colon tissue specimens were obtained from consenting patients at the time of partial colectomy. For the research described here, colon tissue came from a 56-year-old female patient with ischemic colitis. The colon tissue was washed in PBS, cut into small fragments (0.5 x 0.5 cm) and treated with PBS or EPICERTIN (0.1 and 1.0 μM) in complete RPMI 1640 medium for 6 and 12 h, as described previously^21^, before total RNA extraction. A colon tissue fragment at 0 h was embedded in OCT compound for cryosectioning and immunohistochemistry.

#### Statistical analysis

An unpaired, two-tailed Student’s *t*-test was used to compare 2 data sets, whereas 3 or more data sets, were analyzed by one or two-way ANOVA with Bonferroni’s multiple comparison posttest. Data and graphs were analyzed using Prism v.9.1.0 (GraphPad Software, La Jolla, CA, USA). A *p* value of < 0.05 was considered significant.

## Supplementary Material

1

2

3

## Data Availability

The datasets generated during the current study are available from the corresponding author on reasonable request. RNA-seq data from human PBMC-derived macrophages are available in the Gene Expression Omnibus (GEO) database under accession number GSE318500.

## Appendix A. Supplementary data

Supplementary data to this article can be found online at https://doi.org/10.1016/j.mucimm.2026.01.013.
