# Supplementary material for: EPICERTIN, an engineered variant of cholera toxin B subunit, promotes survival and a pro-remodeling macrophage phenotype for mucosal healing in colitis
Source: Mucosal Immunol. Author manuscript; Available in PMC 2026 Apr 29. (PMC13126374; doi:10.1016/j.mucimm.2026.01.013)
Supplement: 3 [file NIHMS2168189-supplement-3.pdf]

Supplementary Material

EPICERTIN, an engineered variant of cholera toxin B subunit, promotes survival and a pro-remodeling macrophage phenotype for mucosal healing in colitis.

Authors: Noel Verjan Garcia[1], Jimmy Fernando Cifuentes[2], Micaela A. Reeves[2], Jae Yeon Hwang[5,6], Juw Won Park[5,6,7], Susan Galandiuk[4] & Nobuyuki Matoba*[1,2,3]

Affiliations

[1]Center for Predictive Medicine, University of Louisville School of Medicine, Louisville, KY, USA

[2]Department of Pharmacology and Toxicology, University of Louisville School of Medicine, Louisville, KY, USA

[3] UofL Health – Brown Cancer Center, University of Louisville School of Medicine

[4] Department of Surgery, Price Institute of Surgical Research, University of Louisville, Louisville, Kentucky, USA

[5]The Center for Integrative Environmental Health Sciences Biostatistics and Informatics Facility Core, University of Louisville, Louisville, KY, USA

[6]Brown Cancer Center Bioinformatics Core, Department of Medicine, University of Louisville, KY, USA

[7]KY INBRE Bioinformatics Core, University of Louisville, Louisville, KY, USA

***Correspondence:** Nobuyuki Matoba, University of Louisville School of Medicine, 505 S. Hancock Street, Room 615, Louisville, KY 40202, USA, Tel: (502) 852 8412; Fax: (502)852 5468; E-Mail: n.matoba@louisville.edu

Supplemental Figures.

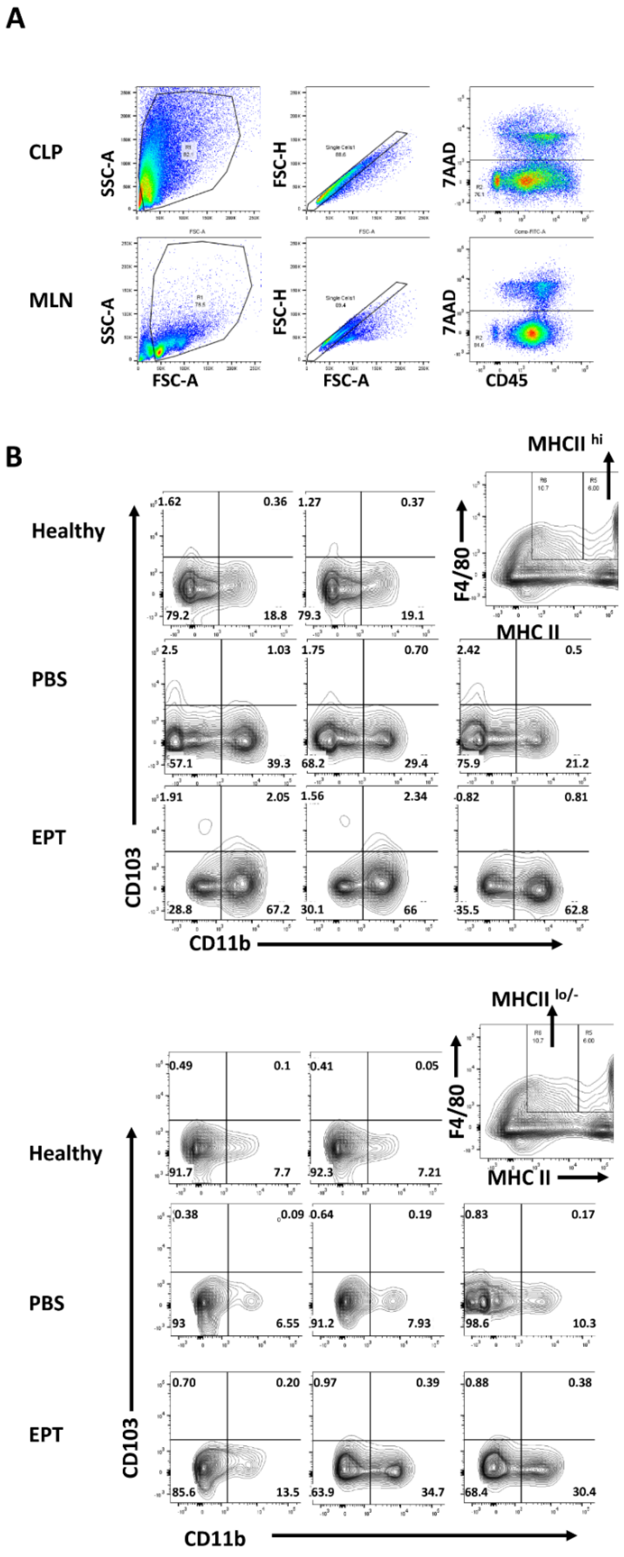

C

DENDRITIC CELLS

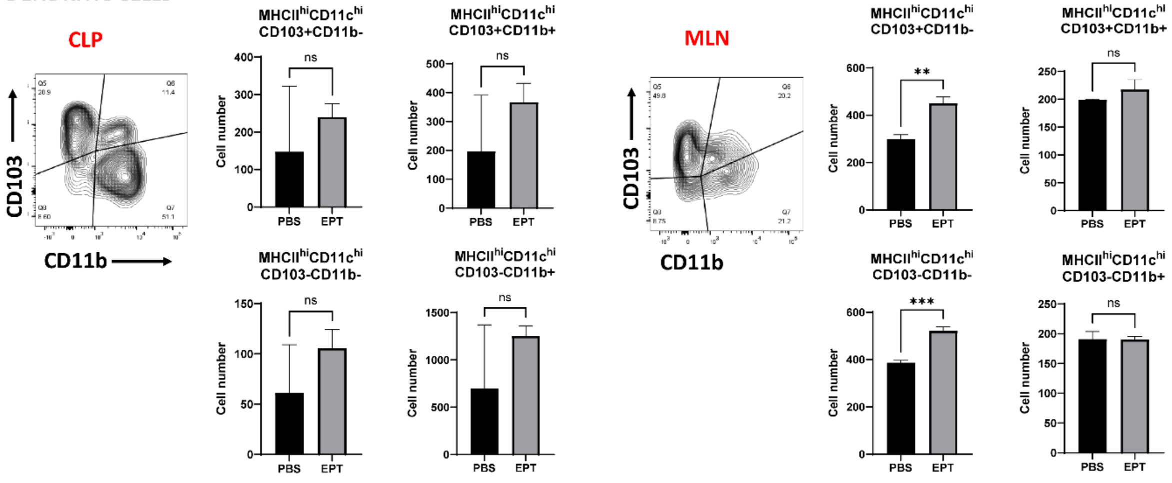

D

LYMPHOCYTES

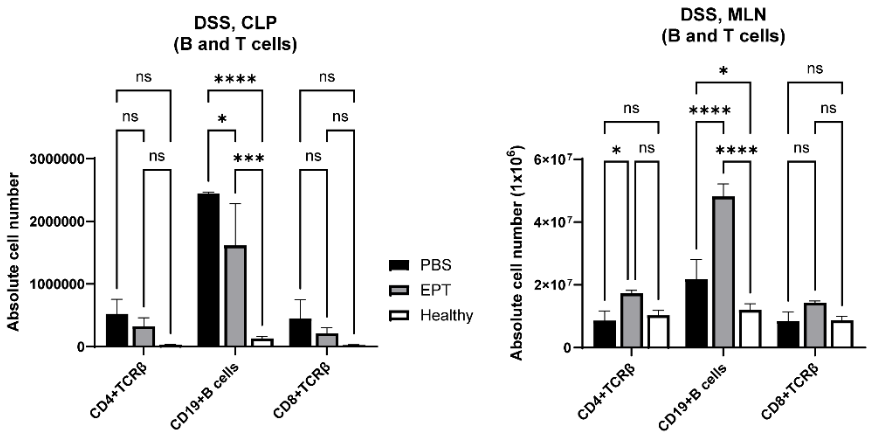

Supplemental Figure 1. ***EPICERTIN recruits more F4/80+ CD11b+ MHCII$^{lo/-}$ M2-like than M1-like macrophages into the lamina propria of DSS-colitis mice.*** **A**. Gating strategy of a representative experiment used to identify M1-like and M2-like macrophages from the colon lamina propria of DSS colitis mice treated with PBS, EPICERTIN or in healthy mice. DSS was administered in the drinking water for 7 days to induce colitis, and mice were treated with PBS or two doses of 3 µg EPT orally (day 3 and 6). Isolated colon lamina propria and mesenteric lymph node leukocytes, such as MHCII$^{hi/lo}$ macrophages (**B**), dendritic cells (**C**) and lymphocytes (**D**) were analyzed by flow cytometry three days after the last dose. An unpaired, 2-tailed Student's t test was used to compare two datasets, whereas One-way ANOVA with Bonferroni's multiple comparison test was used to compare 3 or more groups. Significant differences are indicated with asterisks (*$p < 0.05$, **$p < 0.01$, ***$p < 0.001$, ****$p < 0.0001$).

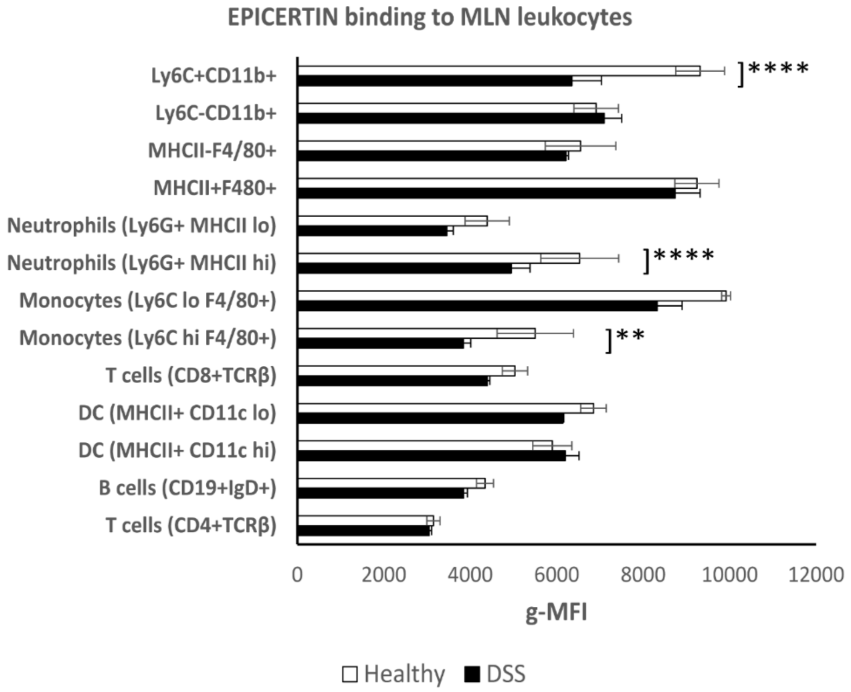

Supplemental Figure 2. ***EPICERTIN binds preferentially to myeloid cells than lymphoid from mesenteric lymph nodes and binding decreases upon inflammation.*** Binding of EPICERTIN to mesenteric lymph node leukocytes from healthy and DSS colitis mice. Binding of EPICERTIN is high in myeloid cells compared to lymphocytes and binding decreases upon inflammation. EPICERTIN bound more to Ly6C+CD11b+ than Ly6C-CD11b+ and more to MHCII+ than to MHCII- myeloid cells. One-way ANOVA with Bonferroni's multiple comparison test was used to compare between groups. Significant differences are indicated with asterisks (*p < 0.05, **p < 0.01, ***p < 0.001, ****p < 0.0001).

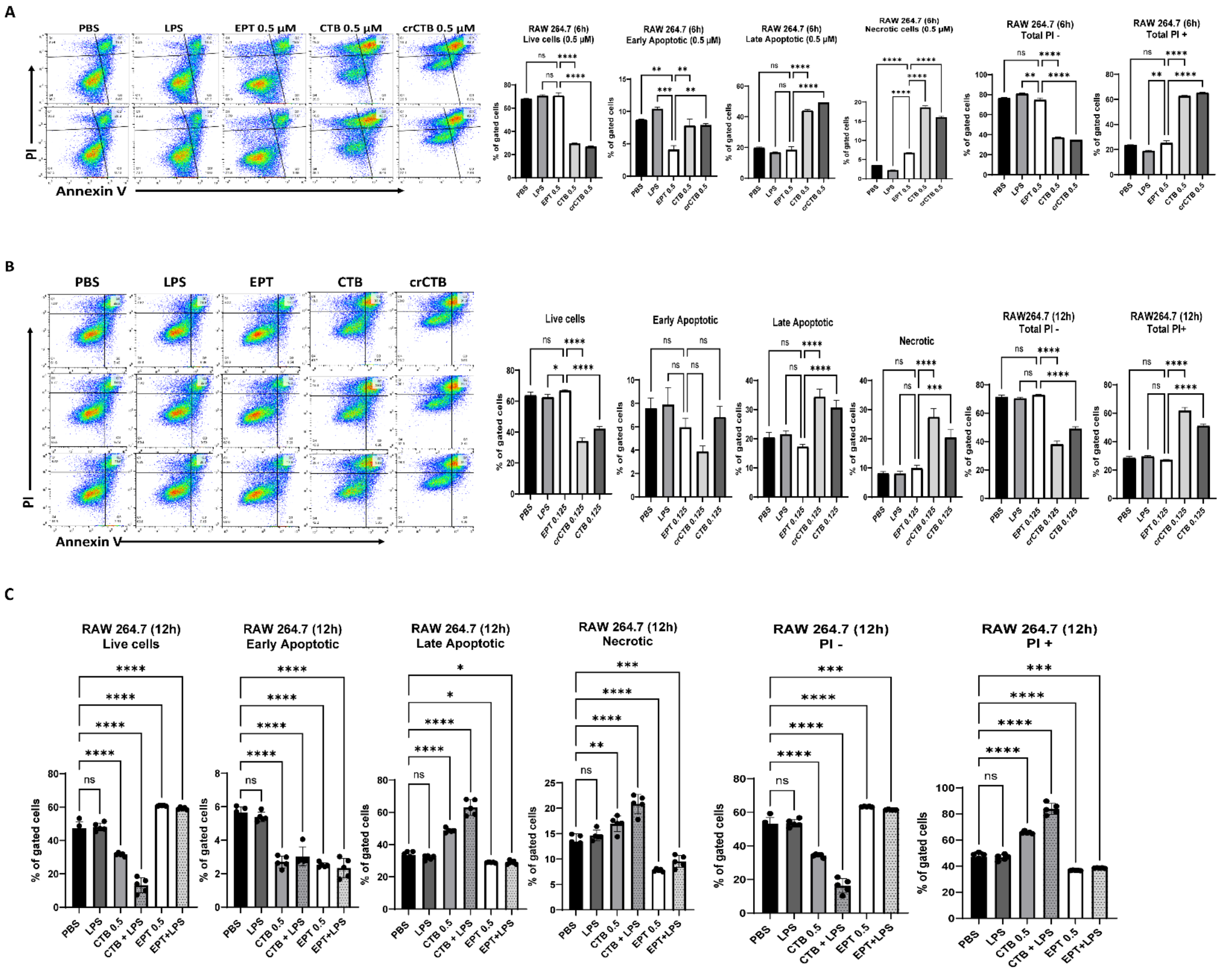

Supplemental Figure 3. Proapoptotic effects of CTB in RAW264.7 macrophages are independent of the protein production system but synergize with *E. coli* LPS O111:B4. The effects of EPICERTIN and CTB on RAW264.7 macrophages were compared to the effects of a commercial recombinant CTB (crCTB) produced in HEK293 cells (Sigma Aldrich) for 6 h (**A**) and 12 h (**B**). A low or high dose (0.125 or 0.5 μM) EPICERTIN promoted cell survival, and the high dose significantly reduced the frequency of early apoptotic cells (p<0.001), whereas crCTB induced a significant (p<0.0001) increase in late apoptotic and necrotic RAW264.7 cells that was slightly higher than that observed with our inhouse CTB produced in *E. coli.* **C.** EPICERTIN treated RAW264.7 macrophages (0.5 μM) promoted a significant increase (p>0.0001) in live cells and reduced both late apoptotic and necrotic cells regardless of the presence of LPS O111:B4. CTB on the other hand, significantly reduced live cells and increased late apoptotic and necrotic cells which synergistically increased in the presence of LPS O111:B4. One-way ANOVA with Bonferroni's multiple comparison test was used to compare between groups. Significant differences are indicated with asterisks (*p < 0.05, **p < 0.01, ***p < 0.001, ****p < 0.0001). A representative experiment from two is shown in plots and each data point represents the mean ± SD of duplicate (**A**) triplicates (**B**) or five (**C**) replicate samples.

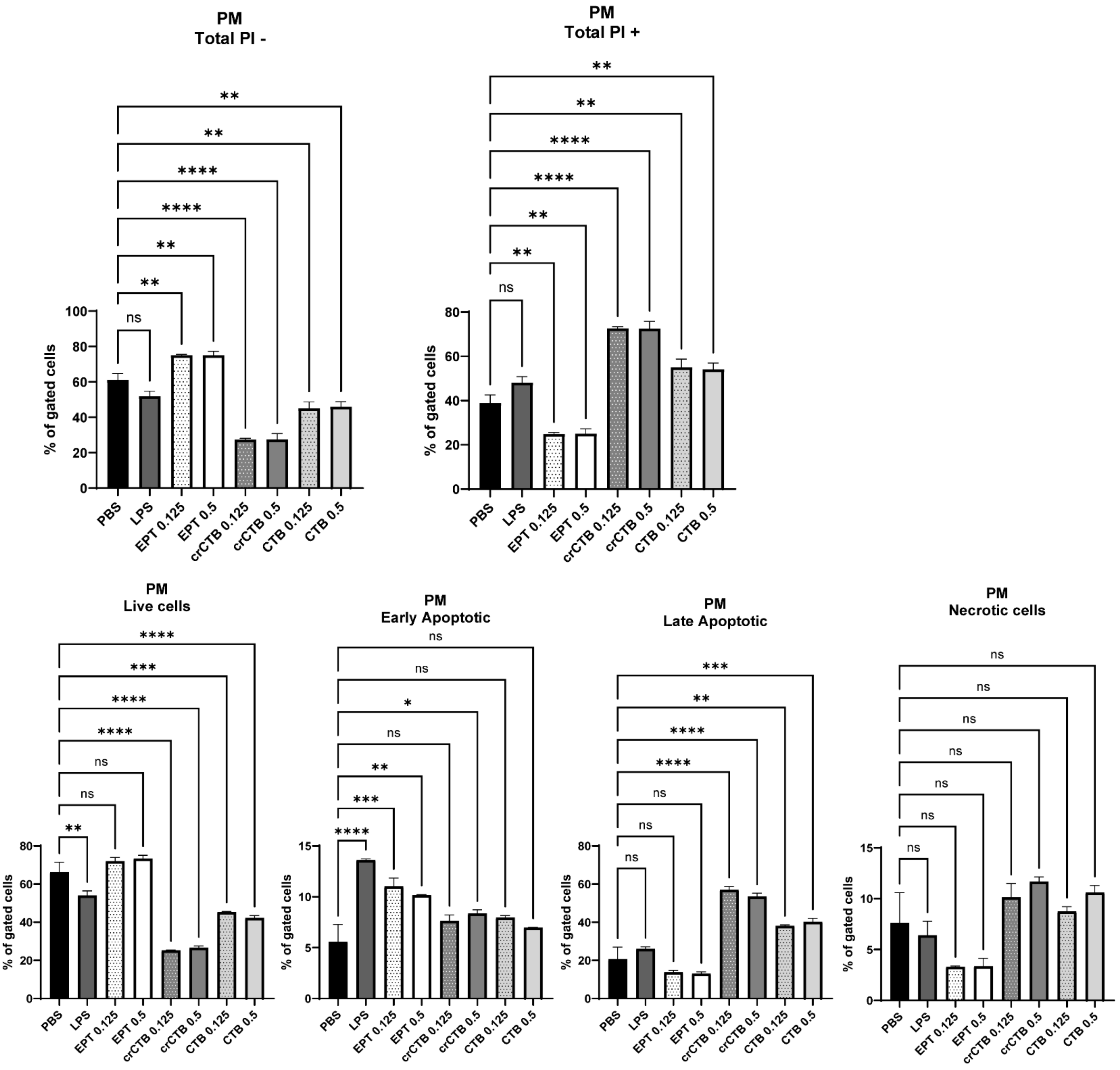

Supplemental Figure 4. EPICERTIN promotes survival of resident peritoneal macrophages, whereas CTB induces apoptosis independent of the protein production system. Peritoneal macrophages (PM) from healthy C57BL/6 mice were treated with a low (0.125 µM) and a high (0.5 µM) dose of EPT or CTB for 12 h in serum-free medium. EPICERTIN promoted the survival of primary peritoneal macrophages with no significant differences compared to PBS control. In contrast, crCTB produced in HEK293 cells or in *E. coli* induced a significant increase in late apoptotic, necrotic and early apoptotic cells or in total PI+ cells. EPICERTIN also showed about 5% increase in early apoptotic cells. One-way ANOVA with Bonferroni's multiple comparison test was used to compare between groups. Significant differences are indicated with asterisks (*$p < 0.05$, **$p < 0.01$, ***$p < 0.001$, ****$p < 0.0001$).

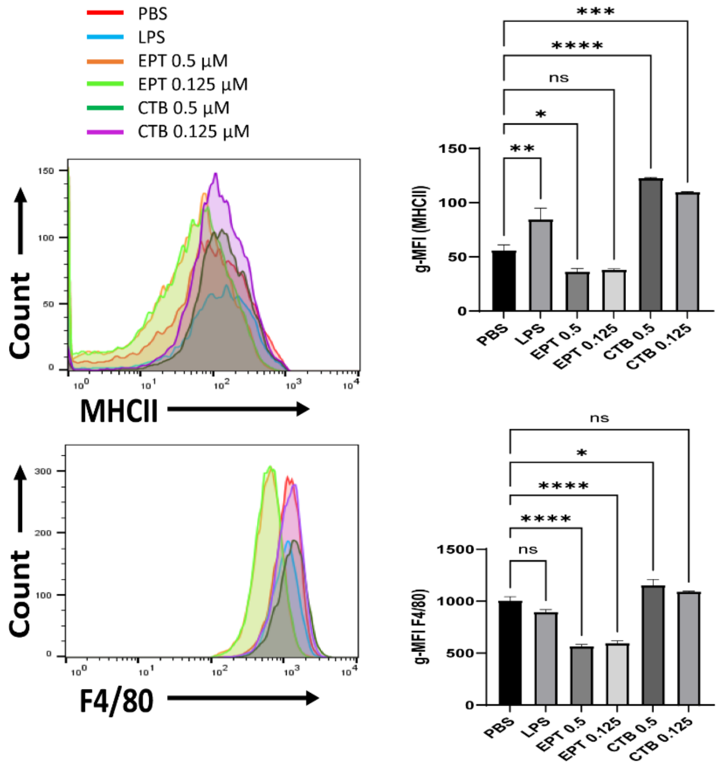

Supplemental Figure 5. EPICERTIN and CTB differ in modulating cell surface molecules on mouse macrophages. EPICERTIN decreases whereas CTB increases MHCII and F4/80 molecules on the surface of resident peritoneal macrophages of mice. Peritoneal macrophages from C57BL/6 mice were treated with PBS, LPS R515 (1 µg/mL), EPICERTIN (EPT on graphs) or CTB at the indicated doses (0.125 and 0.5 µM) for 24 h to analyze cell surface levels of MHCII and F4/80 molecules by flow cytometry. EPICERTIN significantly reduced the surface levels (gMFI) of both MHCII and F4/80 molecules, whereas CTB significantly increased them regardless of the dose. One-way ANOVA with Bonferroni's multiple comparison test was used to compare between groups. Significant differences are indicated with asterisks (*$p < 0.05$, **$p < 0.01$, ***$p < 0.001$, ****$p < 0.0001$.

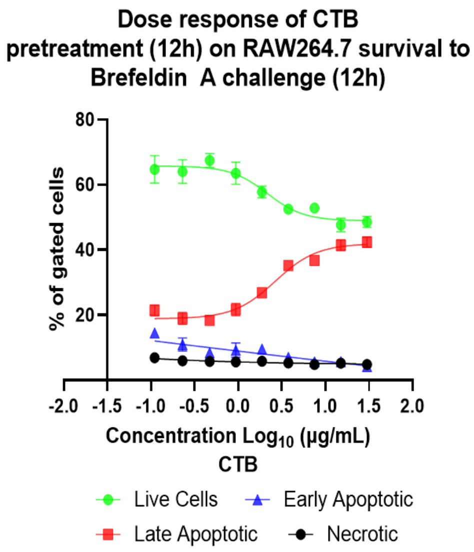

Supplemental Figure 6. Pretreatment with CTB exacerbates brefeldin A-induced apoptosis of RAW264.7 macrophages. RAW264.7 macrophages were cultured in serum-free medium and pretreated with various doses of CTB for 12 h. Subsequently Brefeldin A was added to the medium for an additional 12 h before Annexin V/Propidium iodine staining. The estimated EC50 value for CTB effect on reducing live cells and increasing apoptotic cells was 2.1 µg/mL (1×10^6 cells).

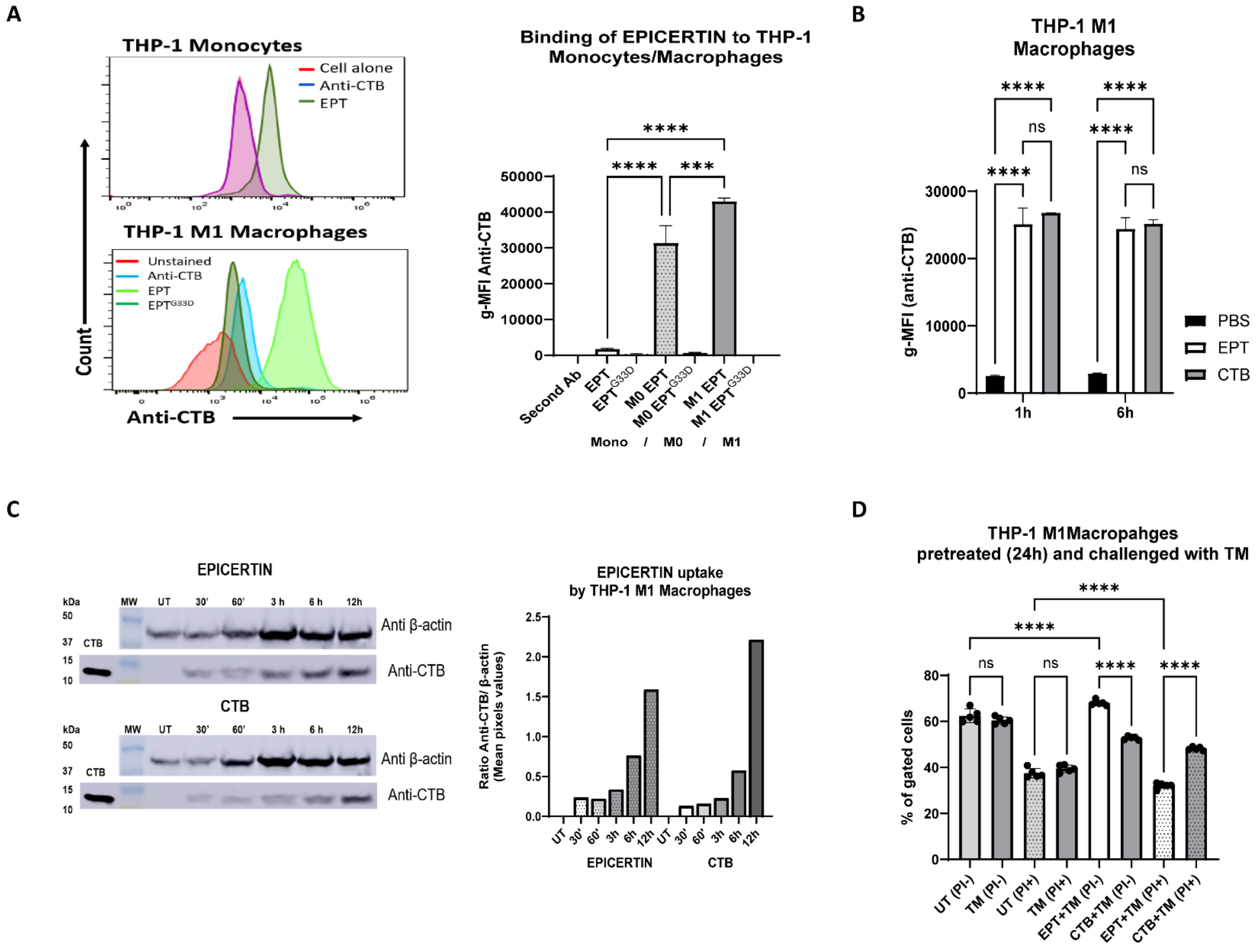

Supplemental Figure 7. EPICERTIN and CTB distinctively activate human THP-1 macrophages. A. Binding of EPICERTIN to human THP-1 monocytes, M0 macrophages (PMA-stimulated), and M1 (hIFN-γ + LPS-polarized) increased after maturation, differentiation or polarization. **B.** Human THP-1 M1 macrophages treated with EPICERTIN or CTB for 1 and up to 6 h show similar levels of surface-bound proteins. **C.** THP-1 M1 macrophages (1×10^6 cells /mL) were cultured in complete RPMI 1640 medium in the presence of EPICERTIN or CTB (0.25 μM) for up to 24 h and cell lysates were prepared at the indicated time points to detect bound internalized proteins by Western blot using 9F9C7 mAb. A similar pattern of internalized EPICERTIN and CTB is shown in THP-1 M1 macrophages. **D.** THP-1 M1 macrophages were pretreated with EPICERTIN or CTB (0.25 μM) for 24 h and challenged with 5 μg/mL Tunicamycin for an additional 24 h before Annexin V/PI staining of live and apoptotic cells. Tunicamycin did not have significant effects on apoptosis (PI + cells) or live cells (PI -) when used at 5 or 10 μg/mL. CTB significantly reduced live cells while increasing dead cells. EPICERTIN significantly increased live cells ($p<0.0001$) while decreasing dead cells. One-way ANOVA with Bonferroni's multiple comparison test was used to compare between groups. Significant differences are indicated with asterisks (*$p < 0.05$, **$p < 0.01$, ***$p < 0.001$, ****$p < 0.0001$).

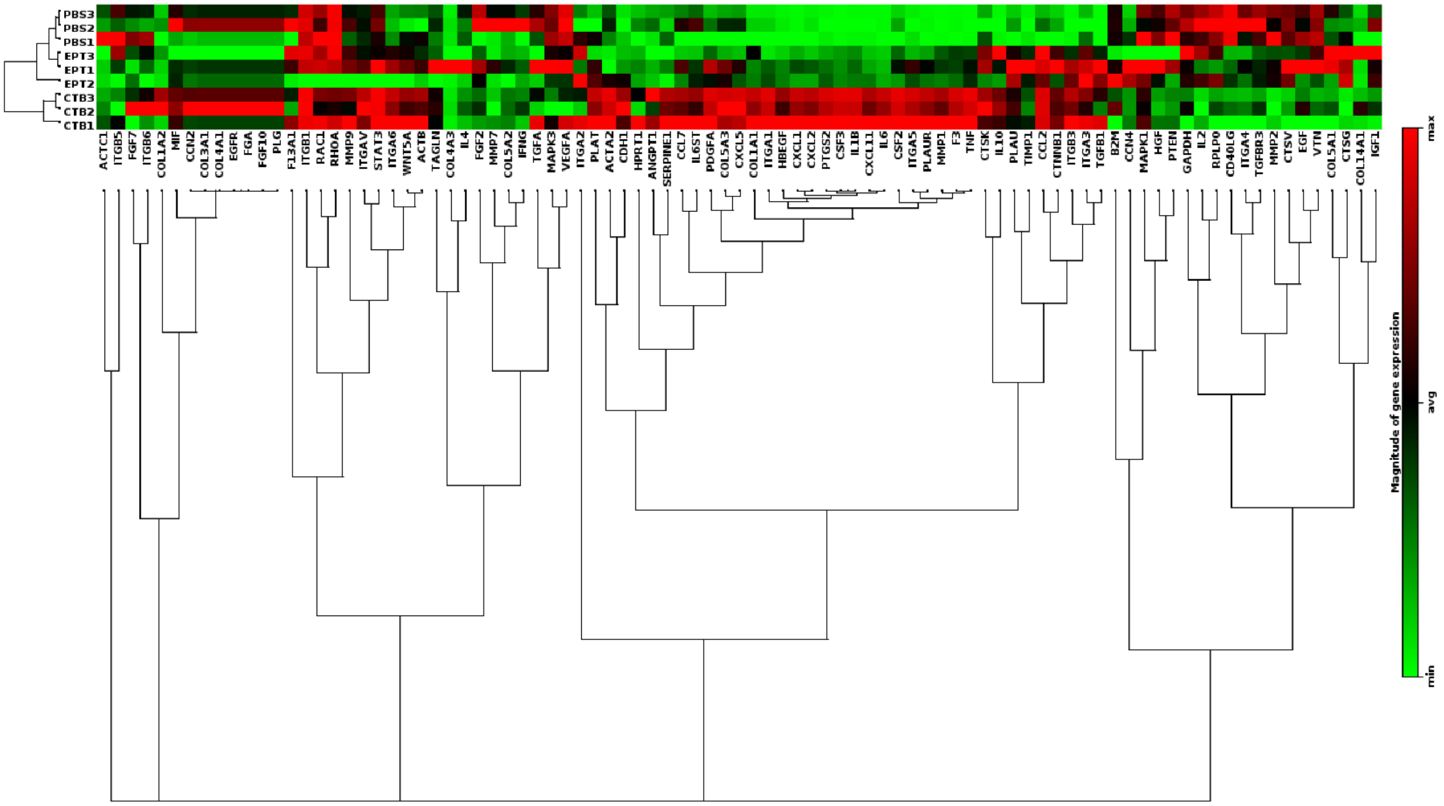

Supplemental Figure 8. Clustergram of wound healing associated genes modulated by EPICERTIN and CTB in human PBMC-derived macrophages. Human PBMC-derived macrophages were generated from human PBMC treated with recombinant human M-CSF (CSF1, 50 ng/mL) in complete RPMI for 6 days in 6-well plates and subsequently treated with EPICERTIN, CTB or PBS for 12 h in RPMI 1640 medium supplemented with 2% FBS. Total RNA was extracted with Trizol and 1 µg used in qRT-PCR analysis of wound healing genes using a human wound healing qRT-PCR array. The level of EPICERTIN induced wound healing gene transcription clustered separately from CTB and close to PBS treatments upon normalization with the glyceraldehyde 3-phosphate dehydrogenase (GAPDH) gene. Analysis was conducted with the Qiagen Gene Globe Data Analysis Center (Qiagen inc., https://geneglobe.qiagen.com/us/analyze).

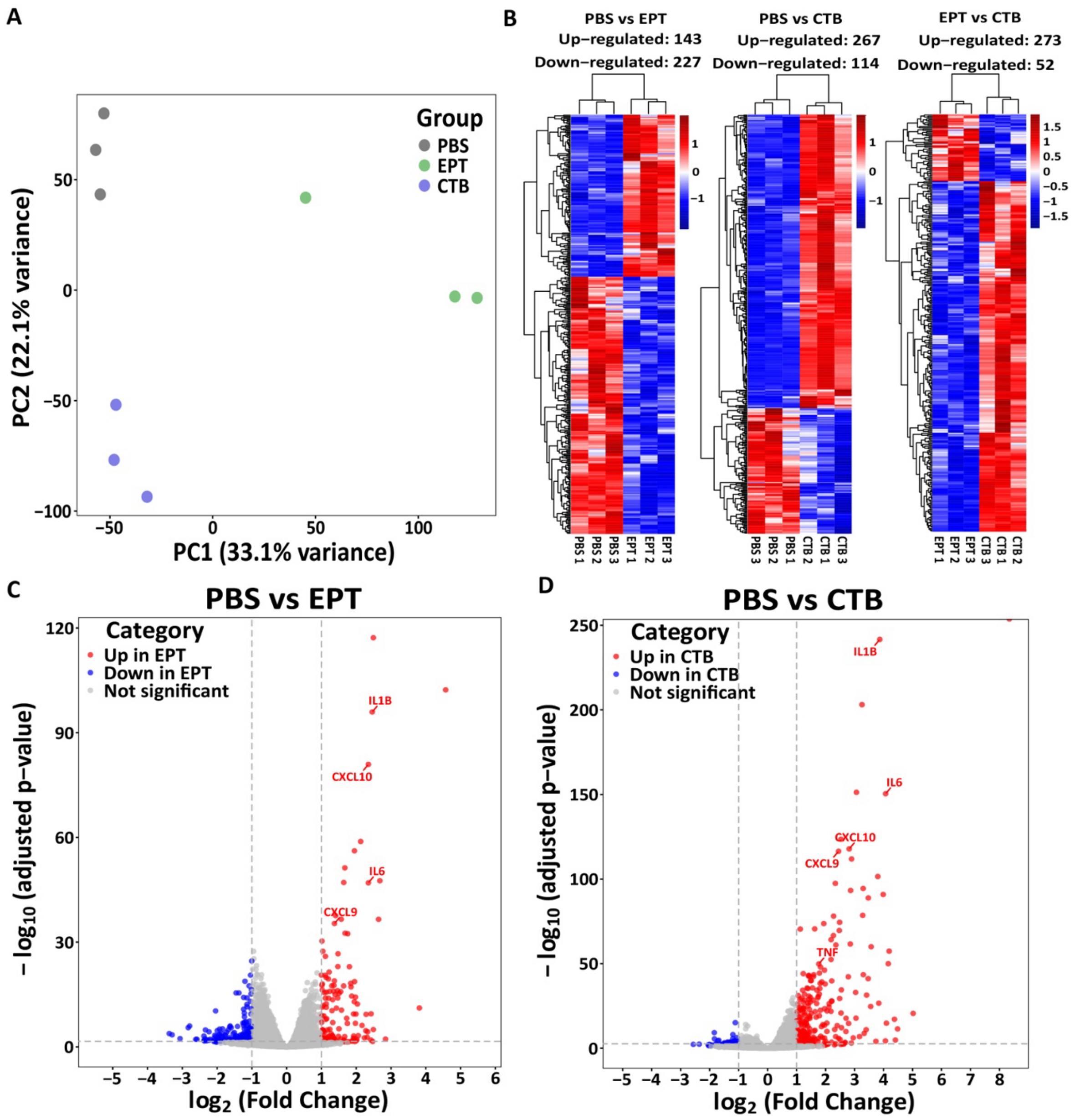

Supplemental Figure 9. Transcriptomic profiling reveals distinct gene expression patterns following EPT and CTB exposure. Robust inflammatory transcriptional activation in response to EPT and CTB treatment, with partially overlapping but distinct gene expression signatures. **A.** Principal Component Analysis of normalized gene expression shows clear separation of the three sample groups: PBS (gray), EPT (green), and CTB (blue), indicating distinct global transcriptional responses. **B.** Expression Heatmaps of significantly differentially expressed genes (DEGs) for each pairwise comparison: PBS vs EPT, PBS vs CTB, and EPT vs. CTB. DEGs were selected using adjusted p-value < 0.05 and $|\log_2 \text{ fold change}| \geq 1$. The number of upregulated and downregulated genes for each comparison is indicated above the heatmaps. Scaled Z-score expression values are shown with scale. **C.** Volcano Plot showing DEGs between PBS vs EPT. Upregulated genes in EPT (red), downregulated genes in EPT (blue), and non-significant genes (gray) are shown. Key proinflammatory-related genes (e.g., *IL1B*, *CXCL10*, *IL6*, *CXCL9*) are labeled. **D.** Volcano Plot displaying DEGs between PBS vs CTB, showing strong induction of proinflammatory-related genes, including *IL1B*, *IL6*, *CXCL10*, *CXCL9*, and *TNF*.

Supplemental Methods

Reagents

Recombinant cholera toxin B-subunit (crCTB; SAE0069) produced in HEK293 cells were purchased from Sigma (Sigma Aldrich). LPS from *E. coli* serotype R515 and Phorbol 12-myristate 13-acetate were from Enzo Life Sciences, Inc (Farmingdale, NY). LPS O111:B4 and LPS O55:B5 were from Millipore Sigma. Recombinant human IFN-γ were from PEPROTECH® (Cranbury, NJ). Recombinant human M-CSF (CSF1) was from Proteintech (Rosemont, IL, USA) or from Gibco (ThermoFisher Scientific). Human cryopreserved peripheral blood mononuclear cells (PBMC) were from Hema Care Charles River Company (Northridge, CA) or STEMCELL TECHNOLOGIES INC. (Kent WA, USA). Lamina Propria Dissociation Kit mouse from Miltenyi Biotec (Bergisch Gladbach, Germany). PE-conjugated mouse anti-human HLA-DR were from BD Biosciences (Sparks, MD). Alexa Fluor 647- anti mouse/human CD324 (E-cadherin), AF-594 anti-F4/80, AF-594 anti-Ki67, AF-647 anti-MHCII (IA-IE) were obtained from Biolegend. AF-488-conjugated Annexin V and Propidium iodide were from Biotium, Inc (Fremont, CA) or from BD Pharmingen™ (BD Biosciences). Anti-CTB (ab34992) was from Abcam (Cambridge, UK). FITC-9F9C7 mAb was reported previously.[1]

SDS-PAGE and Western blot analysis of macrophage cell lysates

RAW264.7 or THP-1 M1 (1×10^6 cells /mL) macrophages were cultured in complete DMEM or RPMI 1640 media, respectively, in the presence of PBS, EPICERTIN or CTB (1 μM) for the indicated time points. Cell pellets were lysed in 2× SDS-PAGE sample buffer followed by boiling at 100 °C for 10 min. Total proteins were separated in 12.5 % polyacrylamide precast gels and transferred to PVDF membranes using the Trans-Blot® Turbo™ transfer system (BIO-RAD). PDVF membranes were blocked with PBST containing 3% non-fat dry milk and incubated with anti-CTB 9F9C7 primary mAb at 2 μg/mL for 1 h at RT. HRP conjugated Goat-anti-Rat IgG (Southern Biotech 3030-05) secondary antibody was used at 1:15000 dilution and incubated for 1 h at RT. Beta actin was detected with an HRP-conjugated (BA3R) mAb diluted 1:1000 (ThermoFisher Scientific). PDVF membranes were incubated with the chemiluminescence detection reagent (Amersham ECL Prime) and the images were analyzed using Fiji software.

CLP, MLNs and peritoneal cavity immune cell isolation

CLP leukocytes were isolated using the Miltenyi Lamina propria dissociation kit mouse and the gentleMACS Dissociator (Miltenyi Biotec). Briefly, the colons were washed, and 0.5 cm long tissue fragments were incubated in predigestion solution (Ca^{2+}, Mg^{2+} free 1x HBSS) containing 10mM HEPES, 5mM EDTA, 5% FBS, and 1 mM DTT at 37 °C for 20 min under continuous rotation to remove epithelial cells and intraepithelial lymphocytes. The colon tissue fragments were digested in digestion solution (1 x HBSS containing Ca^{2+}, Mg^{2+}, 10 mM HEPES and 5% FBS) containing enzymes D, R, and A and incubated for 20 min at 37 °C in the gentleMACS Dissociator using the LPDK_1 program. Cell suspensions were passed through 40 µm cell strainers and CLP leukocytes were counted and used in flow cytometry. MLN were minced in FACS buffer to prepare a single cell suspension. Peritoneal cells were collected by washing the peritoneal cavity using PBS containing 5 % FBS and 5 mM EDTA. The cells were counted and used in flow cytometry or cultured in complete DMEM to allow adherence for in vitro studies.

Flow cytometry

Cells were counted and the Fc receptors were blocked with 10% FBS in PBS containing 20 µg/mL of mouse γ-globulins (Rockland, Limerick, PA). The cells were incubated with 5 µg/mL CTB, EPICERTIN or an EPICERTIN variant with Gly33→Asp mutation (EPICERTIN^{G33D}) for 30 min on ice. After two washes with FACS buffer, FITC-labeled anti-CTB 9F9C7 mAb or fluorochrome-labeled antibodies to cell specific markers including APC-conjugated anti-CD19 (1D3) from eBioscience, anti-CD45eFluor450 or anti-CD45-FITC (30-F11), anti-CD3-FITC or APC (17A2), anti-CD161 (NK1.1)-BV605 (PK136), anti-CD49b-PE (DX5), anti-CD107-AlexaFluor700 (1D4B), anti-CD335 (NKp46)-BV650 (29A1.4), anti-CD16.2-PE-Dazzle 594 (9E9), anti-CD11b-APC-Cy7 (M1/70), anti-CD11c-PE or APC (N418), anti-IA-IE-BV421 (M5/114.15.5), anti-F4/80-PE-Cy7 or APC (BM8), anti-Ly6G-APC (1A8), anti-Ly6C-AlexaFluor700 or PE (HK1.4), anti-CX3CR1-BV605 (SA011F11), anti-CD206-BV650 (C068C2), anti-CD103-PE-Dazzle 594 (QA17A24), anti-CD80-BV605 (16-10A1), anti-CD69-BV650 or FITC (H1.2F3), anti-CD68-AlexaFluor700 (FA-11), anti-CDC86-PE-Dazzle 594

(GL-1), anti-CD4-BV605 or PE (RM4-5), anti-CD8-BV650 or APC (53-6.7), anti-IFNγRβ-APC (MOB-47), anti-IL-33R-PE-Dazzle 594 (DIH4), anti-CD62L-APC-Cy7 (MEL-14), anti-TCRβ-PE-Cy7 (H57-597), anti-IL23R-BV421 (12B2B64) and anti-TCRγ/δ-PE (UC7-13D5), anti-IA-IE-PE (M5/114.15.6), all from Biolegend, were added at 2 µg/mL and the cell suspension incubated on ice for 30 min. After two washing steps the cells were incubated with 7-aminoactinomycin D for 15 minutes and analyzed with a BD SLRFortessa™ or BD FACS Symphony A1 (BD Biosciences) flow cytometer. The data were processed with FlowJo_v10.8.0_CL software.

Immunocytochemistry of macrophages

Peritoneal macrophages (5×10^5 cells/well) were allowed to adhere to 8-well chamber slides for 3 h at 37 °C and non-adherent floating cells were removed. Macrophages were treated with CTB, EPICERTIN, or LPS at the indicated concentrations or PBS in serum-free DMEM supplemented with 0.1% BSA for 3 h. Macrophages were fixed in 100% Methanol at -20 °C for 4 min and stained with FITC-conjugated anti-CTB (9F9C7) mAb, Alexa Fluor 594 anti-F4/80 mAb, and AF-647 anti-MHCII (IA-IE) mAb at 2 µg/mL. Apoptotic macrophages were detected by Click-iT™ Plus TUNEL assay (ThermoFisher Scientific). Duolink® In Situ Mounting medium containing DAPI (Millipore SIGMA) was used to stain the nuclei, and the images were collected with a Nikon A1R Confocal laser scanning microscope using 20 × and 60 × magnification lenses with appropriate channels and the data processed with the NIS Elements imaging software.

Immunohistochemistry of frozen colon tissues

Colon tissues from human patients with ulcerative colitis or colon tissues from EPICERTIN (3, 6 or 10 µg/100µL) treated mice were washed with PBS before embedding in OCT compound. Cryosections (7 µm thick) were processed by IHC as previously described [1]. Briefly, cryosections were fixed in Methanol and stained with AF-647- anti mouse/human CD324 (E-cadherin), AF594 anti-F4/80, AF594 anti-Ki67, AF647 anti-MHCII (IA-IE) or PE-conjugated anti-HLA-DR and anti-CTB (FITC-9F9C7 mAb) at 2 µg/mL of for 1 hr at RT. Nuclei were stained with DAPI and images were collected and analyzed by confocal laser scanning microscopy as described above.

RNA isolation

Human colon tissue explants from ulcerative colitis patients were sectioned into approximately 0.5 × 0.5 cm pieces and treated with EPICERTIN or PBS in serum-free EMEM for the indicated time points. Human colon explants or colon tissues from DSS colitis mice were homogenized in Trizol or Qiazol reagents using a Precellys homogenizer for total RNA isolation using the RNeasy Easy mini-Kit from Qiagen Sciences, Inc (Germantown, MD). RNA concentration, quality, and purity were confirmed by spectrophotometer and then RNA was stored at -20°C until use.

Quantitative RT-qPCR

First strand cDNA was reverse transcribed from 500 ng – 1μg RNA using the Qiagen Sciences Inc, RT^2 First strand kit. Template cDNA was added to the RT^2 SYBR Green Rox qPCR Master Mix and loaded into a Human Wound Healing RT^2 Profiler PCR Array or a Mouse Wound Healing RT^2 Profiler PCR Array using Standard 96-well plates (Qiagen). Gene quantification was carried out on a QuantStudio 3 Real-Time PCR System (Thermo Fisher Scientific) with the following conditions: 1 cycle (10 min at 95 °C); 40 cycles (15 s at 95 °C; 1 min at 60 °C). The Qiagen GeneGlobe web-based platform was used to analyze the CT values, normalization of target vs. housekeeping genes in treated vs. control samples, and the reported fold regulation values of each gene were used to make graphs.

RNA sequencing (RNA-seq) analysis of human PBMC-derived macrophages

Human PBMC-derived macrophage differentiation and treatment – Recombinant human M-CSF (CSF1) was obtained from Proteintech (Rosemont, IL, USA) or Gibco (Thermo Fisher Scientific). Cryopreserved human peripheral blood mononuclear cells (PBMCs) were purchased from HemaCare, a Charles River Company (Northridge, CA), or STEMCELL Technologies (Kent, WA, USA). PBMC-derived macrophages (M0) were

generated by culturing PBMCs in complete RPMI medium supplemented with 50 ng/mL recombinant human M-CSF for 6 days in 6-well plates. M0 macrophages were then treated with EPICERTIN, CTB, or PBS (n = 3 biological replicates per group) for 12 h in RPMI 1640 containing 2% FBS. Following treatment, total RNA was extracted for sequencing.

Total RNA isolation – Cells were homogenized in TRIzol or QIAzol using a Precellys homogenizer, and RNA was isolated using the RNeasy Mini Kit (Qiagen Sciences, Germantown, MD). RNA concentration, purity, and integrity were assessed using a spectrophotometer, and RNA was stored at −20°C until further processing.

Data Processing and Differential Expressed Gene Analysis – RNA-seq libraries were prepared using the Illumina Stranded mRNA Library Prep Kit. RNA purity, quantity, and integrity were assessed using a GE Nanovue Plus spectrophotometer, Qubit Fluorometer (ThermoFisher, Q32855) with Qubit RNA HS assay Kit, and Agilent Bioanalyzer with RNA Nano 6000 Kit (agilent, 5067-1511), respectively. Each library was prepared from 100 ng of input RNA in 25 μL nuclease-free water. Libraries underwent an additional AMPure XP cleanup to remove adapter dimers, and library size and concentration were validated using the Agilent TapeStation. Final libraries were pooled and sequenced on an Illumina NextSeq 2000 using a P3-100 cycle XLEAP cartridge (Illumina, 20100990) to generate 101 bp single-end reads with dual 10 bp index reads. FASTQ files were produced using BaseSpace DRAGEN (v4.2.7).

Raw read counts ranged from 36.4 million to 51.3 million reads per sample. Processed reads were aligned to the human reference genome GRCh38 (hg38) using STAR[2] (v2.7.11b) in end-to-end mode with Ensembl Release 110 (hg38_v44_Ensembl110) annotation. Gene-level quantification of uniquely aligned reads was performed using featureCounts[3] (v2.1.1).

Downstream analyses were performed in R (v4.4.1) using the DESeq2 package[4] (v1.44.0). Genes with low read counts were filtered prior to differential expression analysis. Differentially expressed genes (DEGs) were selected with adjusted p-value < 0.05 and $|\log_2(\text{fold change})| \geq 1$.

Principal component analysis (PCA) was performed on variance-stabilizing transformed (VST) counts. Heatmaps of significant DEGs were generated using hierarchical clustering of Z-score-transformed expression values.

Volcano plots were created for each pairwise comparison by plotting $\log_2$ fold change against $-\log_{10}$ (adjusted p-value). Significance was shown by thresholds (adjusted p-value) < 0.05 and $|\log_2 (\text{fold change})| \geq 1$ for up- and down-regulated genes.

Supplementary Material References

1. Verjan Garcia N, Santisteban Celis IC, Dent M, Matoba N. Characterization and utility of two monoclonal antibodies to cholera toxin B subunit. *Sci Rep* 2023; **13**(1)**:** 4305.

2. Dobin A, Davis CA, Schlesinger F, Drenkow J, Zaleski C, Jha S *et al.* STAR: ultrafast universal RNA-seq aligner. *Bioinformatics* 2013; **29**(1)**:** 15-21.

3. Liao Y, Smyth GK, Shi W. featureCounts: an efficient general purpose program for assigning sequence reads to genomic features. *Bioinformatics* 2014; **30**(7)**:** 923-930.

4. Love MI, Huber W, Anders S. Moderated estimation of fold change and dispersion for RNA-seq data with DESeq2. *Genome Biol* 2014; **15**(12)**:** 550.
